# Non-responder phenotype reveals apparent microbiome-wide antibiotic tolerance in the murine gut

**DOI:** 10.1038/s42003-021-01841-8

**Published:** 2021-03-09

**Authors:** Christian Diener, Anna C. H. Hoge, Sean M. Kearney, Ulrike Kusebauch, Sushmita Patwardhan, Robert L. Moritz, Susan E. Erdman, Sean M. Gibbons

**Affiliations:** 1 Institute for Systems Biology, Seattle, WA USA; 2 Department of Biological Engineering, Massachusetts Institute of Technology, Cambridge, MA USA; 3 Broad Institute of MIT and Harvard, Cambridge, MA USA; 4 Division of Comparative Medicine, Massachusetts Institute of Technology, Cambridge, MA USA; 5 eScience Institute, University of Washington, Seattle, WA USA; 6 Department of Bioengineering, University of Washington, Seattle, WA USA

**Keywords:** Microbiome, Risk factors, Microbiota, Microbial ecology

## Abstract

Broad spectrum antibiotics cause both transient and lasting damage to the ecology of the gut microbiome. Antibiotic-induced loss of gut bacterial diversity has been linked to susceptibility to enteric infections. Prior work on subtherapeutic antibiotic treatment in humans and non-human animals has suggested that entire gut communities may exhibit tolerance phenotypes. In this study, we validate the existence of these community tolerance phenotypes in the murine gut and explore how antibiotic treatment duration or a diet enriched in antimicrobial phytochemicals might influence the frequency of this phenotype. Almost a third of mice exhibited whole-community tolerance to a high dose of the *β*-lactam antibiotic cefoperazone, independent of antibiotic treatment duration or dietary phytochemical amendment. We observed few compositional differences between non-responder microbiota during antibiotic treatment and the untreated control microbiota. However, gene expression was vastly different between non-responder microbiota and controls during treatment, with non-responder communities showing an upregulation of antimicrobial tolerance genes, like efflux transporters, and a down-regulation of central metabolism. Future work should focus on what specific host- or microbiome-associated factors are responsible for tipping communities between responder and non-responder phenotypes so that we might learn to harness this phenomenon to protect our microbiota from routine antibiotic treatment.

## Abstract (web summary)

Diener, Hoge et al. show that a third of mice exhibit tolerance to a high dose of the *β*-lactam antibiotic cefoperazone, independent of antibiotic treatment duration or dietary phytochemical amendment. They find that non-responder microbiota upregulates antimicrobial tolerance genes and downregulates central metabolism without altering community composition or diversity, providing insights into the mechanisms of community-wide antibiotic tolerance.

## Introduction

Despite the clear public health utility of antibiotics, there is an undeniable cost to their widespread use in medicine and agriculture^1^. Antibiotic resistance in pathogens is on the rise and evidence is mounting that antibiotic treatments cause both transient and lasting damage to our commensal microbiota^2–4^. The gut microbiome is an integral component of the human body, helping with nutrient absorption, pathogen resistance, and immune system education^2^. When the ecology of the gut is compromised by antibiotics, host health can suffer^5–9^.

Previous work in humans has shown that one round of antibiotic treatment can temporarily alter the taxonomic composition of the gut microbiome, increase the prevalence of antibiotic resistance genes, and lead to a long-term reduction in species diversity^10–16^. The steady decline of gut bacterial diversity in developed nations over the last century, likely due in part to antibiotic use, has been implicated in the rise of chronic immune dysfunction and severe nosocomial infections^3,15,17–21^. Thus, finding ways to prevent or mitigate the ecological damage done by antibiotics is an important public health priority^15,21^. For example, strategies have been developed to introduce activated carbon into the lower gut during antibiotic exposure to protect colonic bacteria^22^ or to use autologous fecal transplants to replenish gut diversity following treatment^23^. In addition to these therapeutic strategies, the microbiome appears to exhibit natural antibiotic tolerance under certain conditions. The same sub-therapeutic doses of antibiotics in animal models have been shown to substantially reduce gut microbiome diversity and biomass in some hosts but not in others, indicating that these gut communities vary in their capacity for tolerance^24–26^. In single-strain systems, sub-populations of antibiotic-resistant cells arise spontaneously due to stochastic apportionment of efflux transporters between daughter cells^27,28^ or due to the spontaneous amplification of antimicrobial resistance genes in mutant sub-populations^29^ (i.e. spontaneous “symmetry-breaking” processes within otherwise homogeneous populations of cells). Analogous symmetry-breaking processes^27,28,30^ may contribute to observed community-level antibiotic tolerance within the microbiome^24,26^.

Heterogeneous responses of gut microbiota to therapeutic antibiotic treatments have been reported in the literature^12,24,31,32^. For example, while antibiotic exposure is a risk factor for *Clostridioides difficile* carriage and infection in hospitals, not all antibiotic-treated patients exposed to *C. difficile* become infected^18,19^. Certain classes of antibiotics, like fluoroquinolones and cephalosporins, are more likely to increase *C. difficile* infection risk in humans^21^. To investigate this phenomenon in a more controlled system, Schubert et al.^33^ looked into how the type and concentration of antibiotic treatment influenced *C. difficile* colonization of the murine gut. The authors built a Random Forest regression model that could accurately predict *C. difficile* colonization levels from the composition of the gut microbiome. Cefoperazone, a third-generation cephalosporin and broad-spectrum *β*-lactam antibiotic, had a large and fairly consistent effect on the composition of the gut microbiome across most mice, lowered bacterial biomass by up to three orders of magnitude, and made mice more susceptible to *C. difficile* colonization and infection^33^. Indeed, cefoperazone-treated mice have become an accepted experimental platform for studying *C. difficile* colonization and infection^34^. Interestingly, certain mice that received relatively high doses of cefoperazone in Schubert et al.^33^ were not colonized by *C. difficile* following exposure. These mice were also not predicted to be colonized by the RF model and thus appeared to maintain a gut microbiome composition that was similar to the control mice. Based on these results, we hypothesized that whole-community antibiotic tolerance to a high dose of cefoperazone might be a common phenomenon in the murine gut.

In this study, we explore the associations between bacterial community composition, community-wide gene expression, and community-wide cefoperazone tolerance, and we look at how the prevalence of this tolerance phenotype (hereafter referred to as the “non-responder” phenotype) varies across certain treatment regimes. Although it was not a focus of their work, Schubert et al.^33^ showed that the frequency of the non-responder phenotype decreased with higher concentrations of cefoperazone, which comports with prior work on sub-therapeutic antibiotic treatments in mice^24,25^. Other important factors that could influence the frequency of this non-responder phenotype, which we set out to test, were the duration of antibiotic exposure^35^ and prior dietary exposures to antimicrobial phytochemicals^36–39^. We designed and carried out two independent mouse experiments to explore the reproducibility and frequency of non-responders to a high dose of cefoperazone (100–150 mg/kg/day)^33,34^ across duration and dietary phytochemical treatments. In the duration experiment, we exposed mice to 2, 4, 8, and 16 days of cefoperazone treatment. In the diet experiment, we included a 1% seaweed amendment to normal mouse chow, as used previously by our group^37^. We hypothesized that increased exposure to plant-derived “secondary compounds” (i.e. defensive phytochemicals produced by plants to protect themselves against herbivores and microbial pathogens) might influence subsequent responses to antibiotics^40,41^, and raw seaweed is a rich source of these compounds^42,43^. In addition to measuring community composition and biomass, we sequenced community transcriptomes in non-responder and control microbiomes to characterize the gene expression profiles associated with apparent community-wide tolerance. Finally, we quantified potential heterogeneity in cefoperazone concentrations in blood and stool samples for a subset of mice.

Across both experiments, we found that 31% (10 out of 32) of singly housed mice exposed to the same high dose of cefoperazone, which has been used to promote *C. difficile* colonization in prior mouse studies^33,34^, were protected from antibiotic-induced ecological collapse, independent of duration or dietary treatments. The phylum-level community structure, species diversity, and biomass of these non-responder microbiomes were similar to untreated controls and reproducible across both experiments. Despite minimal change in community composition, non-responder microbiota showed dramatic differences in community transcriptional profiles when compared to untreated mice (>25% of all gene functions were differentially expressed). Gene functions involved in growth and motility were downregulated and antimicrobial efflux transporter genes were upregulated in non-responder microbiomes. On average, cefoperazone concentrations were slightly lower in non-responder fecal samples when compared to responder fecal samples 2 days after the cessation of antibiotic treatment, either due to host heterogeneity or to bacterial metabolism. Together, these results show that entire gut bacterial communities frequently escape ecological collapse during broad-spectrum antibiotic treatment.

## Results and discussion

### Antibiotic duration experiment

**Fig. 1:**
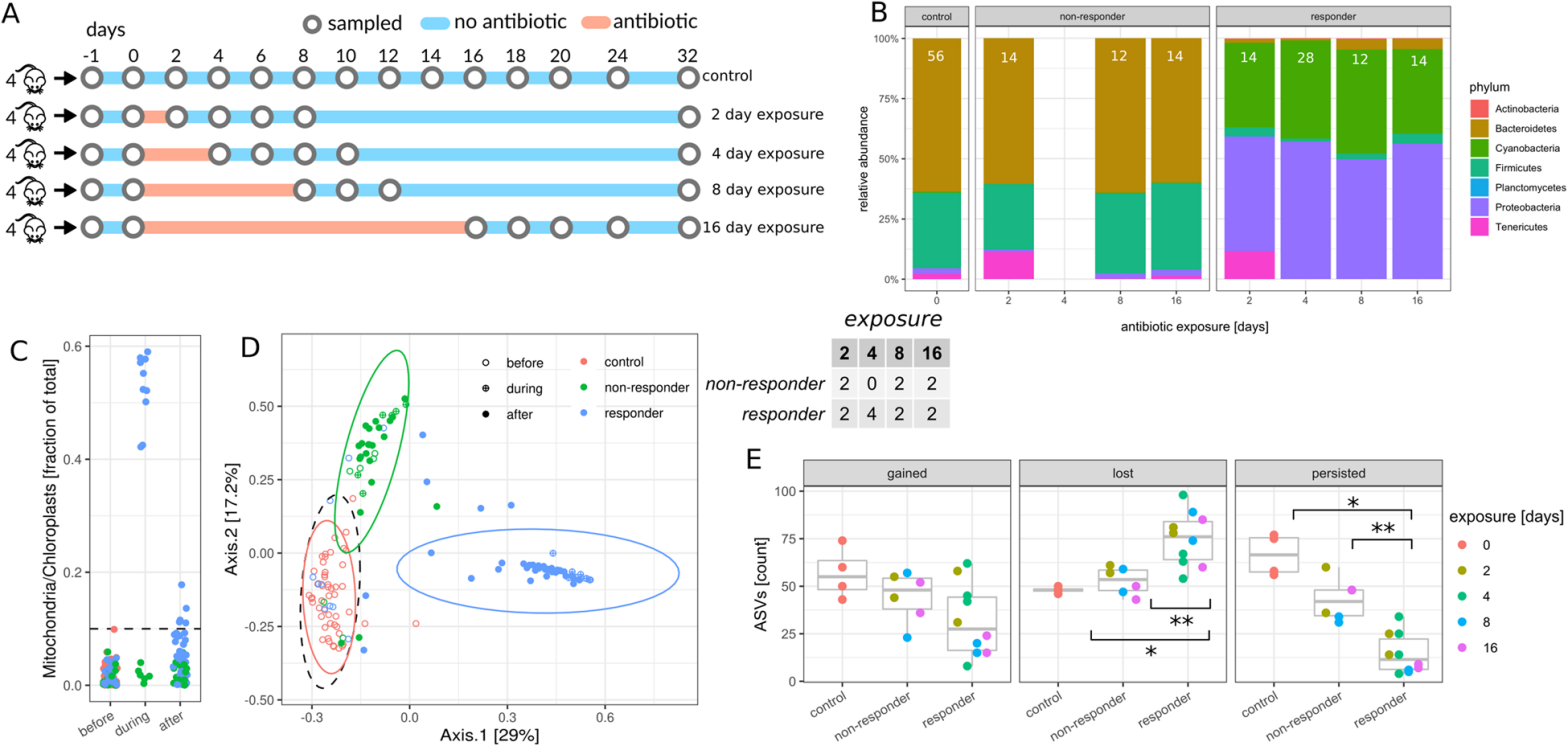
Effect of antibiotic exposure duration on non-responder phenotype. The table in the center denotes the number of non-responder and responder mice in each treatment duration group. **A** Experimental design for the duration experiment. Circles denote sampled time points. Time points were considered sampled “during” antibiotic treatment between day 0 and day 2, 4, 8, and 16, respectively, as denoted by orange shades. **B** Relative abundance of phyla on the last day of antibiotics treatment. The control panel is an average over all untreated controls from all time points. Only phyla with a relative abundance of at least 0.1% are shown. Each barchart denotes means from at least two samples and white insets are the sample size used for each barchart. **C** Percentage of mitochondria and chloroplast sequences in 16S amplicon data relative to antibiotic treatment. Colors: red—controls not treated with antibiotics, green—non-responders, blue—responders. **D** Principal coordinate analysis (PCoA) of samples during and after antibiotic exposure (*n* = 143 samples with >10,000 reads per sample, day ≥ 0). Ellipses denote 95% confidence intervals from a Student *t*-distribution. Each point denotes a sample. ASV abundances were rarefied to 10,000 reads for each sample and percentages in brackets denote the explained variance. Samples with less than 10,000 reads per sample were not included in the analysis. **E** Dynamics of amplicon sequence variants (ASVs). Gained ASVs are variants that were not present before antibiotics treatment but are present after. Similarly, lost ASVs were present before treatment but not after, and persistent ASVs were present before and after. Stars denote significance under a Mann–Whitney *U* test: *p < 0.05, **p < 0.01.

Twenty-eight-week-old female C57BL/6J mice from the same birth cohort were co-housed (5–6 mice per cage) prior to beginning the experiment, and then separated into individual cages 1 week prior to antibiotic treatment. Singly housed mice were exposed to 0.5 mg/mL^33^ cefoperazone in their drinking water for 0, 2, 4, 8, or 16 days (Fig. 1A). Based on the literature, we calculated the minimum dose of cefoperazone based on the mean and standard deviation of water consumption by C57BL/6J mice ($7.7\, \mp 0.3\,{\mathrm{mL}}$ per 30 g of body weight)^44^. If the heaviest mouse in our study (~22 g) consistently consumed water at 2 SD below the mean (i.e. 5.5 of 0.5 mg/mL cefoperazone), they would still receive 125 mg/kg/day of cefoperazone, which is within the therapeutic dosing range for humans (100–150 mg/kg/day; although cefoperazone is administered to humans via intravenous injection)^45^.

Power analyses were performed by sampling from beta-binomial distributions fit to an independent data set and processed with the same protocol as the data presented in this manuscript (see “Methods”). Beta-binomial distributions have recently been shown to recapitulate 16S amplicon data structure well and are highly flexible distributions that can account for the observed overdispersion in microbiome data without making assumptions about taxon–taxon correlations made by Dirichlet-multinomial distributions^46^. The resulting abundance tables were used to evaluate the expected power of beta-diversity and differential taxon abundance tests as a function of sample number and effect size. From the power curves, we concluded that we can reliably detect differences in beta-diversity (PERMANOVA) with an *R*^2^ as low as 0.05 and with as few as five samples per group (Fig. S1). Furthermore, we estimate that taxon abundance differences larger than twofold can be reliably detected with 5–10 samples per group using beta-binomial likelihood ratio test (abbreviated beta-binomial LRTs from here on, Fig. S1). 16S amplicon sequencing of the duration experiment demonstrated that most of the cefoperazone-treated mice showed altered gut microbiome communities during antibiotic treatment (Fig. 1). These mice showed pronounced turnover in community composition at the phylum level, with a near-complete loss of Bacteroidetes and Firmicutes (beta-binomial LRT false discovery rate FDR-corrected *p* < 1e−9) and a dramatic enrichment of Proteobacteria and Cyanobacteria (beta-binomial LRT FDR-corrected *p* < 1e−11; Fig. 1B). SILVA annotations showed that 98% of Proteobacteria and Cyanobacteria reads were identified as being derived from organelles (i.e. mitochondria and chloroplasts). Thus, these reads were likely derived from plant chloroplasts and mitochondria in the diet or host mitochondria. The observed shift towards mitochondrial and chloroplast reads in most of the antibiotic-treated samples is likely a consequence of antibiotic-induced collapse of bacterial biomass, which would increase the detection of the background contaminants, such as host- and diet-derived organelles. However, 6 of the 16 cefoperazone-treated mice in this duration experiment did not exhibit an enrichment in mitochondrial and chloroplast reads during antibiotic exposure (Fig. 1B, C). Thus, the microbiota in these mice appeared to be antibiotic tolerant. Consequently we designated individual mice showing [mitochondria+chloroplast] relative abundance $\geqq 10\%$ (i.e. greater than the highest observed fraction observed in untreated controls) during antibiotic exposure as antibiotic “responders”, whereas mice showing [mitochondria+chloroplast] relative abundance <10% during antibiotic exposure were designated as antibiotic “non-responders”. The only duration where all mice responded to antibiotic treatment was the 4-day exposure (Fig. 1B). Overall, duration of exposure had no significant influence over the frequency of non-responder phenotypes (Fisher’s exact test *p* = 0.44). Prior to antibiotic treatment, there were no significant beta-diversity differences between responder and non-responder mice (PERMANOVA *R*^2^ = 0.06, *p* = 0.37). Additionally, we found no differences in phylum-level abundances between controls, responders, and non-responders before antibiotic exposure (all beta-binomial LRT FDR-corrected *p* > 0.8). The microbiome composition of non-responder mice during exposure was similar to untreated control mice at the phylum level, with only the Tenericutes phylum showing a significantly lower abundance in non-responders (beta-binomial LRT FDR-corrected *p* = 0.007, Fig. 1B).

Initially, we had predicted that duration of exposure would be positively correlated with within-host amplicon sequence variant (ASV) extinction (i.e. ASVs present within a mouse initially, but not at the end of the experiment). To quantify the persistence, gain, or loss of ASVs, we tracked the presence or absence of sequence variants within the same mouse over time. ASVs present within a mouse at the first and final time points of the study were considered to be “persistent”, ASVs present in a mouse at the beginning of the experiment and missing at the last time point were considered “lost”, and ASVs absent at the beginning and present at the end were considered “gained”. Treatment duration had no significant effect on persistence, loss, or gain of ASVs (ANOVA *p* > 0.1). There was a significant increase in the proportion of lost ASVs and a significant decrease in persistent ASVs in responder mice, when compared to untreated control mice (Mann–Whitney *U* *p* < 0.02, Fig. 1E). Non-responder mice, however, showed no significant differences from controls in ASVs gained, lost, or persistent (all Mann–Whitney *U* *p* > 0.1, Fig. 1E). Thus, non-responder microbiota were apparently protected from phylum-level collapse of gut bacterial community structure and ASV loss following antibiotic treatment.

### Seaweed diet experiment

**Fig. 2:**
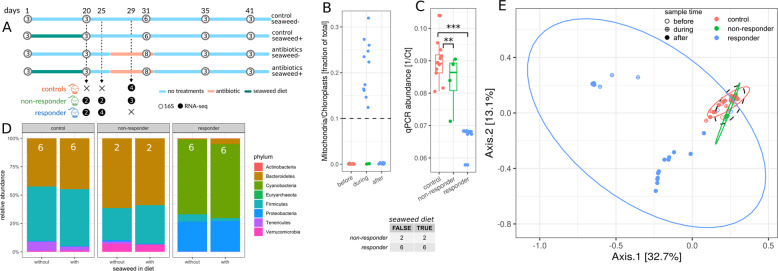
Effect of seaweed diet on non-responder phenotype. The table in the center shows the number of non-responder and responder mice in each diet group. **A** Design of the diet experiment. White circles denote 16S samples and are filled with the number of biological replicates for each sampling point. Black circles denote RNA-seq samples. **B** Percentage of mitochondria and chloroplast sequences in 16S amplicon data relative to antibiotic treatment. Colors: red—controls not treated with antibiotics, green—non-responders, blue—responders. **C** qPCR biomass estimates (1/Ct) for samples across response groups during antibiotics exposure. *N* = 12, 4, and 12 for controls, non-responders, and responders, respectively. Stars denote significance under a Mann–Whitney *U* test: ***p < 0.001, **p < 0.01. **D** Relative phyla abundances across diet and response groups. Each barchart denotes means from at least two samples and white insets are the sample size used for each barchart. Only phyla with a relative abundance larger than 0.1% are shown. Colors: red—controls not treated with antibiotics, green—non-responders, blue—responders. **E** PCoA of 16S samples after diet (*n* = 60 samples with more than 5000 reads, day ≥ 20). Symbol fill denotes sampling time relative to antibiotic treatment and colors denotes response type. Ellipses denote 95% confidence interval from a Student’s *t*-distribution. Dashed ellipse describes samples taken before antibiotic exposure. ASV abundances were rarefied to 5000 reads for each sample, and four samples with fewer than 5000 reads were not included in the analysis. Percentages in brackets show explained variance by that axis.

Twenty-eight seven-week-old female C57BL/6J mice from the same birth cohort were co-housed prior to beginning the experiment (5–6 mice per cage), and then separated into individual cages 1 week prior to dietary treatments. Mice were assigned to four treatment groups: eight mice in group A (seaweed−/antibiotic+), six mice in group B (seaweed−/antibiotic−), eight mice in group C (seaweed+/antibiotic+), and eight mice in group D (seaweed+/antibiotic−). We included a larger number of replicate mice in the antibiotic treatment groups because we wanted to capture as many non-responders as possible. Half of the mice (treatment groups C and D) were given a 1% seaweed in normal chow diet and the other half (groups A and B) received a normal chow diet for 20 days (Fig. 2A). All mice were put on the same normal chow diet for six days prior to antibiotic treatment to rule out any potential direct effects of seaweed on antibiotic absorption during antibiotic treatment (e.g. potential binding and inactivation of the antibiotic by seaweed-derived compounds). On day 26, all mice continued on a normal chow diet and mice in treatment groups A and C were given 0.5 mg/mL cefoperazone in their drinking water for a period of 6 days, with mice in groups B and D acting as untreated controls (Fig. 2A). We hypothesized that prior exposure to antimicrobial secondary metabolites in red seaweed, such as polyphenols, bromophenols, and terpenes^37–39,47^, may “harden” the gut microbiota against future damage from antimicrobials, and perhaps promote tolerance of cefoperazone. Stool pellets from the full set of replicate mice were sampled each day and frozen at −80 °C in glycerol with 0.1% l-cysteine (16 antibiotic-treated mice and 12 control mice) and a subset of these samples were processed for sequencing (Figs. 2A and S2).

**Fig. 3:**
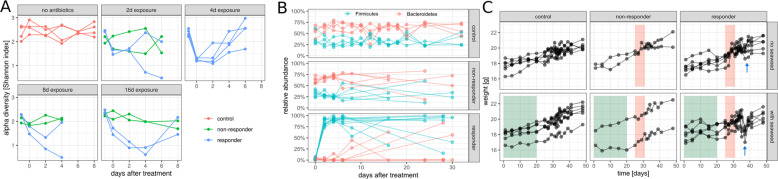
Temporal dynamics in non-responder and responder mice following antibiotic and diet treatments. **A** Alpha diversity (Shannon index) dynamics after antibiotics treatment in the duration experiment. Each point denotes a single sample and samples from the same mouse are connected by lines. Colors denote responder status. **B** Dynamics of Bacteroidetes and Firmicutes phyla in the antibiotic duration experiment. **C** Mouse weights in the diet experiment. Green areas denote seaweed diet treatment windows and red areas denote antibiotics treatment windows. The blue arrows indicate transient weight loss in responder mice a few days following the end of antibiotic treatment (day 35–40). Responders vs. controls Mann–Whitney *U* *p* = 0.03 (*n* = 48) and seaweed diet responders vs. normal chow responders *p* = 0.02 (*n* = 24).

Seaweed treatment had a very minor impact on the composition and diversity of mouse gut microbiomes (Fig. S2), similar to what we had observed previously^37^. We identified the same non-responder and responder phenotypes as in the duration experiment, with 4 of the 16 mice exhibiting the non-responder phenotype (i.e. $\geqq 10\%$ [mitochondria + chloroplast]; Fig. 2B). The seaweed diet had no effect on the frequency of the non-responder phenotype (Fisher’s exact test *p* = 1.0). To validate that the observed enrichment in organelle relative abundances indeed corresponded to a lower bacterial biomass, we measured total 16S gene copy numbers in each sample (i.e. a proxy for bacterial biomass; Fig. 2C). As expected, 16S copy number was inversely proportional to the fraction of mitochondria and chloroplasts (Spearman rho = −0.84, *p* = 2.7e−8). In particular, we found that responder mice showed lower fecal bacterial biomass following cefoperazone treatment than controls (Mann–Whitney *U* *p* = 4.4e−6) and non-responders (Mann–Whitney *U* *p* = 0.004), while non-responder microbiomes did not differ significantly from controls in biomass levels during antibiotic exposure (Mann–Whitney *U* *p* = 0.6, Fig. 2C). Thus, it indeed appears that the absence of appreciable bacterial biomass in a mouse gut results in an enrichment for host and dietary contaminants in stool 16S amplicon sequencing data.

We observed similar phylum-level compositions between controls and non-responders during antibiotic exposure, with a slight decrease in Bacteroidetes and a loss of Tenericutes in non-responders (beta-binomial LTR FDR-corrected *p* < 2e−3), and small differences in beta-diversity between controls and non-responder samples during treatment (*R*^2^ = 0.07, PERMANOVA *p* = 0.03, all beta-binomial LRT *p* < 2e−3, Fig. 2D, E). As mentioned above, responder mice showed the same transition to Cyano- and Proteobacteria dominance and were well-separated from controls and non-responders in beta-diversity space (*R*^2^ = 0.2, PERMANOVA *p* = 0.001, Fig. 2D, E). Seaweed treatment did not affect beta-diversity (*R*^2^ = 0.01, PERMANOVA *p* = 0.3).

To exclude potential differences between responders and non-responders prior to antibiotic treatment, we sequenced an additional set of 44 archived fecal samples in a second batch, which included 16 non-responder, 16 responder, and 12 control samples, before and during antibiotic exposure (see “Methods”, Fig. S3). We observed no significant difference in beta-diversity between controls, responders, and non-responders prior to antibiotic treatment (PERMANOVA *R*^2^ = 0.12, *p* = 0.14). Again all sample groups were similar at the phylum level prior to treatment (all beta-binomial LRT *p* > 0.36), with some slight variation in the abundance of the *Erysipelatoclostridium* genus between groups (beta-binomial LRT *p* = 0.02). Consistent with our prior data, we saw a large shift in beta-diversity during treatment between responders and non-responders (*R*^2^ = 0.41, PERMANOVA *p* = 0.001, Fig. S3). Thus, the compositional shift in phylum abundances we observed was indeed triggered by antibiotic treatment and could not be explained with pre-existing differences in the microbiome composition (Fig. S3). Interestingly, one mouse initially classified as non-responder (A4) based on data from day 31 clustered with responders on day 29 (Fig. S3), which suggests that responders are able to transition to non-responder phenotypes during the course of antibiotic treatment.

We determined the abundance of cefoperazone in a subset of stool and blood plasma samples by Selected Reaction Monitoring (SRM), a targeted, quantitative mass spectrometry technique (Fig. S4). We observed that cefoperazone reached concentrations well above its observed MIC_50_ for a wide range of 357 anaerobic bacterial strains^48^ in responder mice during antibiotic treatment (Fig. S4A). Two days post antibiotic treatment, average stool antibiotic concentrations were lower in non-responders, although concentrations remained at similar levels as responders in at least one-third non-responder mice included in the sample subset for cefoperazone quantification (Fig. S4A). During treatment, cefoperazone concentrations in blood plasma were orders of magnitude lower than in stool samples (<10 ng/mL), and we observed a ~3-fold range in blood concentrations across antibiotic-treated responder mice (Fig. S4B). Due to time constraints and animal welfare concerns, blood collection was done by cheek bleed during antibiotic treatment on only the first three replicate mice from each treatment group (A1–3, B1–3, C1–3, and D1–3). Unfortunately, none of these replicate mice ended up being the non-responders. Furthermore, 16S and metatranscriptome data generation was prioritized, and all non-responder stool samples taken during antibiotic treatment were used up to generate DNA and RNA; hence, we lack cefoperazone data on those samples. However, as mentioned above, the concentration of cefoperazone was lower in two-thirds of the analyzed non-responder samples (compared to responder samples) taken 2 days following the end of antibiotic treatment, which suggests that most non-responders (but not all) may experience a reduced concentration of antibiotic in situ (Fig. S4). Whether these phenotypes are driven by host- or microbiome-associated heterogeneity, it is crucial that we understand the frequency and characteristics of this non-responder phenotype, especially in light of the fact that the cefoperazone treatment used in this study is an established experimental model of *C. difficile* infection in mice^33,34^.

### Temporal dynamics following antibiotic treatment

Shannon diversity declined for at least 2 days after antibiotic treatment in responder mice, and continued to decline for up to 4 days depending on the duration of treatment (Fig. 3A). Despite greater loss of species in responder mice (Fig. 1D), overall alpha diversity tended to recover over time in these mice after cessation of antibiotic treatment (2 days after treatment vs. 6–8 days after treatment Mann–Whitney *p* = 0.03, *n* = 18). Non-responder and control mice maintained relatively stable alpha-diversities over time (2 days after treatment vs. 6–8 days after treatment Mann–Whitney *p* = 0.93, *n* = 22), although antibiotic-treated, non-responder microbiota showed slightly lower alpha-diversities than controls (controls vs. non-responders Mann–Whitney *p* = 8e−8, *n* = 80; Fig. 3A). Despite the resilience of Shannon diversity in the responder mice over time, only a small number of these mice showed recovery of Bacteroidetes ASVs (Fig. 3B). In control and non-responder mice, Bacteroidetes was the dominant phylum over the entire time series. However, Firmicutes became the dominant phylum in responder mice following antibiotics, and in many mice there appeared to be a persistent loss of the Bacteroidetes phylum following recovery (Fig. 3B). Seaweed dietary treatment appeared to contribute somewhat to this loss in resilience, with none of the seaweed-fed responder mice showing recovery of the Bacteroides phylum (Fig. S2).

Mouse weights were measured daily in the seaweed diet experiment to ensure that there were no systematic differences in food and water consumption among mice. We saw no evidence for weight loss in antibiotic-treated mice relative to controls during antibiotic treatment, which would have suggested reduced water consumption (Wald test *p* = 0.17 and 0.4 for non-responders and responders, respectively). Furthermore, we saw no significant difference in weight change between responder and non-responder mice (Wald test *p* = 0.07), suggesting that patterns of food and water consumption were similar between these groups. While we saw no major differences in the gut microbiome structure between control diet (i.e. normal chow) mice and 1% seaweed-fed mice (Fig. 2C and Fig. S1), we did observe a difference in mouse weight loss several days after the end of antibiotic treatment (Fig. 3C). All responder mice showed a transient weight-drop between day 35 and 40 (Mann–Whitney *U* *p* = 0.03 for responders vs. controls; Fig. 3C). This weight-drop was more pronounced in mice-fed seaweed compared to mice on a normal chow diet (Mann–Whitney *p* = 0.02, Fig. 3C). However, none of these mice showed signs of illness or diarrhea. We do not have an explanation for this synergistic effect between seaweed diet and cefoperazone treatment on transient weight loss in mice several days following the cessation of antibiotic treatment, but we believe this to be an interesting research avenue to explore further.

### Non-responder microbiomes exhibit an antimicrobial tolerance transcriptional program

To evaluate whether the occurrence of the non-responder phenotype might be associated with changes in gene transcription in the gut, we performed RNA sequencing on samples from 10 mice before antibiotic treatment (days 20 and 25) and 7 mice during antibiotic treatment (day 29). Because there was almost no bacterial biomass in responder fecal samples during antibiotic treatment (and, consequently, not enough RNA for sequencing), we compared non-responders to untreated controls on day 29. After RNA extraction, ribosomal depletion, and sequencing to a mean depth of 20 million reads per sample, we identified around 800,000 unique transcripts by de novo assembly (see “Methods”) ranging from 111 to >26,000 bp in length (longer contigs were polycistronic; see Fig. S5 for length and coverage distributions).

Power analyses were performed by sampling from negative binomial distributions fit to an independent metatranscriptomic sequencing data set and calculating power with DESeq2 (see “Methods” for more details). Due to the pooled variance inference in DESeq2, differential expression could be detected with as few as six samples (three per group) with an average log2-fold change of 2.5 (uniform between 0 and 5) as long as the mean log2 expression of the transcript was at least 5 (Fig. S1).

**Fig. 4:**
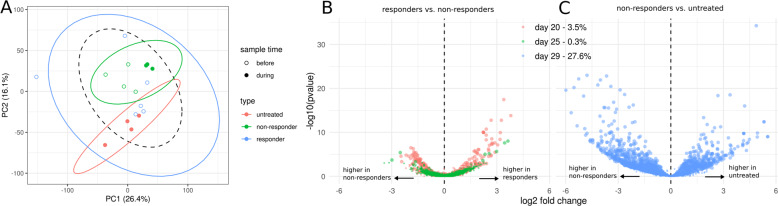
Global transcriptional response to antibiotics in non-responder phenotypes. **A** Principal component analysis (PCA) of RNA-seq samples based on abundances of orthologous protein clusters. Percentages in brackets denotes explained variance. Symbol fill denotes sampling time relative to antibiotic treatment and colors denotes response type. Ellipses denote 95% confidence interval from a Student’s *t*-distribution. Dashed ellipse describes samples taken before antibiotic exposure. **B**, **C** Volcano plots of pre-treatment responder vs. non-responder samples (**B**) and non-responder samples during treatment vs. untreated samples (**C**). Each dot denotes a differential abundance test for a distinct SEED cluster of orthologous proteins. Percentages for each day denote positive tests rate (number of significant tests/total tests) and colors denote the day the samples were taken (days 20 and 25 were before and day 29 was during antibiotic treatment). Tests with FDR *q* values < 0.05 are shown as larger dots, whereas non-significant results are shown as small dots.

Transcripts were collapsed to orthologous protein clusters by alignment to the M5NR database^49^. We were able to assign 61% of the original transcripts to functions in the SEED subsystem database^50^. This allowed us to collapse transcript counts for each sample into SEED clusters, which yielded a total of 53,877 unique orthologous protein clusters. The majority of SEED clusters were detected in all 17 RNA-seq samples (Fig. S6). Control and non-responder communities could be easily distinguished by SEED cluster counts during antibiotic exposure (see Fig. 4A). In particular, about 21% of the variance in functional expression could be explained by non-responder vs. control status (Euclidean PERMANOVA *p* = 0.003) compared to 7% of explained variance in 16S beta-diversity (Bray-Curtis PERMANOVA *p* = 0.03). Thus, transcriptional differences appear to define the non-responder phenotype more than changes in community composition. Prior to antibiotic exposure, responder samples could not be distinguished from control samples (PERMANOVA *p* = 0.3).

After filtering out low abundance functions, differential expression testing between controls and non-responder communities was performed for each of the three time points sampled (see “Methods”). We observed that fewer than 0.5% of the SEED clusters were differentially expressed at an FDR-corrected *p* ≤ 0.05 2 days before antibiotic exposure (day 25), which fits our null-expectation that non-responder and responder mice are functionally similar before exposure (Fig. 4B). However, following antibiotic exposure (day 29), 27% of all SEED clusters were differentially expressed between untreated controls and non-responder communities (see Fig. 4C). This indicated a global transcriptional shift in non-responder microbiomes, mostly characterized by an upregulation of several functional groups in the non-responder mice (blue dots on left side of Fig. 4C).

**Fig. 5:**
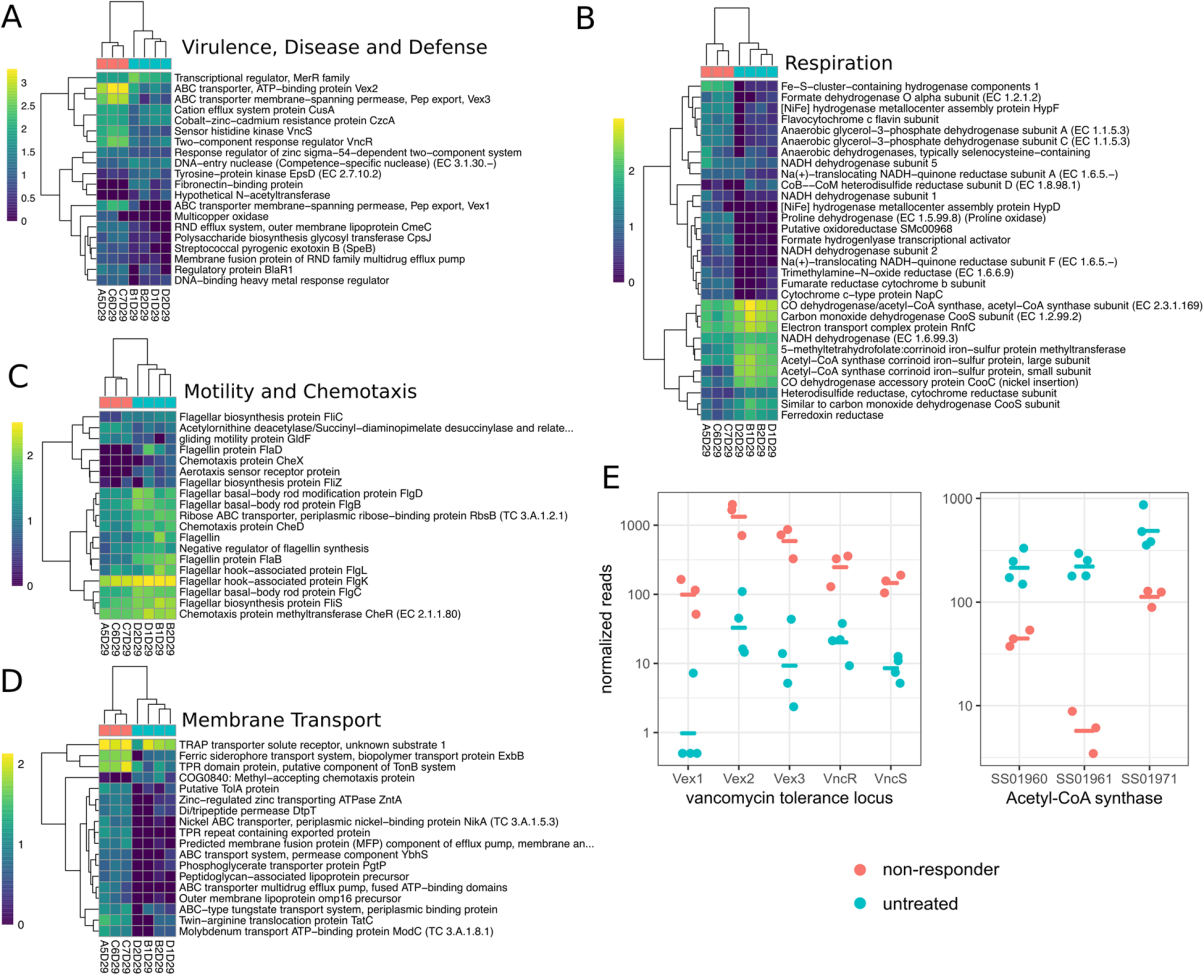
Differentially abundant pathways in non-responder phenotypes. **A**–**D** Heatmaps showing differentially abundant (FDR < 0.05) functional groups grouped by SEED superpathway. Heatmap color scale shows normalized reads on a log10 scale with a pseudocount of 1. Sample names in the columns are a combination of the individual mouse identifiers (e.g. A5 refers to the fifth replicate mouse in the treatment group A) and the day the sample was taken (e.g. D29 refers to day 29). Colors on top of the columns denote response type (red = non-responder, blue = controls). **E** Normalized expression of genes on the vancomycin tolerance locus and three Acetyl-CoA synthase genes between non-responders and controls.

The transcriptional program was most prominently characterized by an upregulation of efflux transporters and other antibiotic resistance defense mechanisms, and a down-regulation of motility and respiratory functions (Fig. 5). For instance, the SEED subpathway “Transporters in Models” was the most prominent subpathway in the differentially expressed functions, containing 82 significant hits (DESeq2 FDR-corrected *p* ≤ 0.05). Most of the significantly upregulated functions in the “Virulence, Disease and Defense” superpathway were also related to efflux pumps and their regulation (Fig. 5A). We also found large differences in respiratory pathways, albeit with a mixed pattern of up- and down-regulation (Fig. 5B). Some of these key respiratory pathways were downregulated by one to two orders of magnitude in the non-responder mice, which suggests an overall down-regulation of ATP synthesis. We found 146 differentially expressed protein clusters associated with carbohydrate metabolism, although these pathways did not show a clear pattern of regulation, with only 74/146 clusters having higher abundances in untreated controls. Additionally, we observed that 17 of the 19 flagellar motor and chemotaxis proteins were downregulated in the non-responder mice (Fig. 5C). All differentially expressed functions in the “Membrane Transport” superpathway were strongly upregulated in the non-responder mice, including components of TonB, which is known to be necessary for efflux transporter function^51^ (Fig. 5D). Together, these data are consistent with previous reports that upregulation of efflux transporters is accompanied by a concomitant reduction in growth rate^27,52^. Indeed, the most striking respiratory difference we observed related to potential growth rate reduction was the down-regulation of three acetyl-CoA synthases, which were some of the most highly expressed functions in the untreated mice (Fig. 5E). Finally, we observed the upregulation of the entire vancomycin resistance locus, including the three efflux pumps Vex1–3 and the two-component system VncR and VncS (Fig. 5E). The induction of vancomycin cross-resistance by *β*-lactams has been described before^53,54^ and might indicate that these loci confer general efflux-based tolerance to a range of antibiotics.

### Conclusion

We found that nearly one-third of mice exposed to a high dose of the cephalosporin antibiotic cefoperazone, commonly used in mouse experiments to promote *C. difficile* colonization^33,34^, did not exhibit the expected gut microbiome community turnover, biomass collapse, or species loss. The frequency of this non-responder phenotype did not depend on duration of antibiotic exposure or on prior exposure to antimicrobial phytochemicals present in seaweed, but did appear to increase as the concentration of cefoperazone in drinking water declined, as shown previously^33^.

Despite very minor changes in community composition and ecological diversity between untreated and non-responder mice, we observe a striking difference in microbiome gene expression between these groups. Non-responder microbiomes show substantial down-regulation of central metabolism and motility, and upregulation of antimicrobial tolerance mechanisms. This combination of increased antibiotic tolerance and quiescence may protect gut communities from the extensive ecological damage observed in responder microbiomes. Furthermore, this may help to protect the gut from pathogen invasion by maintaining a more intact gut microbiota. However, further studies will be required to unravel the potential interplay between non-responder phenotypes and susceptibility to pathogen invasion.

We observed heterogeneity in stool and plasma cefoperazone concentrations, despite no significant differences in mouse weight gain between responders and non-responders (i.e. a proxy for changes in water or food consumption that might have been due to treatment). However, the average cefoperazone concentration in non-responder feces was lower than the average concentration in responder feces two days after antibiotic treatment ended, although one of the non-responder samples still showed cefoperazone levels equivalent to those measured in the responder samples. In one instance, we observed the same mouse switching between responder and non-responder status during the course of antibiotic treatment, which indicates that transitions between these states are possible.

While prior work has shown how isogenic sub-populations of cells and two-species communities can exhibit heterogeneous responses to antibiotics^26–29^, the exact mechanisms underlying transitions to whole-community tolerance phenotypes in the mammalian gut are not yet clear and will require further study. Whether community-wide non-responder phenotypes are driven purely by phenotypic plasticity among isogenic populations of microbes, or whether they arise, in part, due to evolutionary adaptation, remains an open question. Prior work has suggested that antibiotic tolerance can facilitate the evolution of antimicrobial resistance^55^, and exploring whether these non-responder phenotypes enable the fixation of resistance alleles is a topic for future population genomic work. Future work should also focus on identifying what factors tip microbiomes between non-responder and responder phenotypes, potential hysteresis of these phenotypes, and whether or not this transition point can be manipulated to protect commensal microbiota from antibiotic assault.

## Methods

### Animal care

5- to 6-week-old female C57BL/6J mice ordered from Jackson Laboratories (Bar Harbor, ME) were housed and handled in Association for Assessment and Accreditation of Laboratory Animal Care (AAALAC)-accredited facilities using techniques and diets specifically approved by Massachusetts Institute of Technology’s Committee on Animal Care (CAC) (MIT CAC protocol no. 0912-090-15 and 0909-090-18). The MIT CAC (Institutional Animal Care and Use Committee [IACUC]) specifically approved the studies as well as the single-housing and handling of these animals. Mice were euthanized using carbon dioxide at the end of the experiment.

### Antibiotic duration experiment

For this 34-day experiment, 20 mice were assigned randomly and evenly to five treatment groups: control, 2 days of antibiotic exposure, 4 days of antibiotic exposure, 8 days of antibiotic exposure, and 16 days of antibiotic exposure. The *β*-lactam antibiotic cefoperazone was administered through drinking water at a concentration of 0.5 mg/mL, as in prior work^33^. Fecal samples were collected on the 2 days preceding antibiotic exposure, the last day of antibiotic exposure, and select time points following antibiotic exposure. Mice were weighed each sampling day. Fresh fecal samples were obtained within an hour of one another each day from all animals. Fecal samples were collected into 2 mL freezer tubes with 100 μL of anaerobic 40% glycerol containing 0.1% cysteine and transferred immediately to dry ice before being stored at −80 °C prior to nucleic acid extraction.

### Seaweed diet and antibiotic experiment

A new cohort of 28 mice were split randomly into two diet treatment groups and were fed with either a custom chow diet (Bio-Serv, Flemington NJ) containing 1% raw seaweed nori (Izumi Brand) or a standard control diet (product no. F3156; AN-93G; Bio-Serv, Flemington, NJ). Prior to the experiment, animals were co-housed for 10 days and then singly housed for 7 days prior to separation into the seaweed treatment and control groups. After 20 days of dietary treatment, all mice resumed the standard diet. From day 26 to day 31, eight mice from each diet group were administered 0.5 mg/mL cefoperazone in their drinking water as in the duration experiment. All mice were weighed and assessed daily. As per our IACUC protocol, any mouse showing significant signs of morbidity/suffering would be humanely euthanized. No mice showed signs of morbidity or distress during the course of both experiments. Fresh fecal samples were obtained within an hour of one another each day from all animals in all groups. Fecal samples were collected into anaerobic 40% glycerol containing 0.1% cysteine and transferred immediately to dry ice before being stored at −80 °C prior to nucleic acid extraction.

### 16S amplicon sequencing

DNA from fecal samples and bacterial cultures was extracted using the MoBio High Throughput (HTP) PowerSoil Isolation Kit (MoBio Laboratories; now Qiagen) with minor modifications. Briefly, samples were homogenized with bead beating and then 50 μL Proteinase K (QIAGEN) added, and samples were incubated in a 65 °C water bath for 10 min. Samples were then incubated at 95 °C for 10 min to deactivate the protease.

Libraries for paired-end Illumina sequencing were constructed using a two-step 16S rRNA PCR amplicon approach as described previously with minor modifications^56^. The first-step primers (PE16S_V4_U515_F, 5′-ACACG ACGCT CTTCC GATCT YRYRG TGCCA GCMGC CGCGG TAA-3′; PE16S_V4_E786_R, 5′-CGGCA TTCCT GCTGA ACCGC TCTTC CGATC TGGAC TACHV GGGTW TCTAA T-3′) contain primers U515F and E786R targeting the V4 region of the 16S rRNA gene, as described previously^56^. Additionally, a complexity region in the forward primer (5′-YRYR-3′) was added to help the image-processing software used to detect distinct clusters during Illumina next-generation sequencing. A second-step priming site is also present in both the forward (5′-ACACG ACGCT CTTCC GATCT-3′) and reverse (5′-CGGCA TTCCT GCTGA ACCGC TCTTC CGATC T-3′) first-step primers. The second-step primers incorporate the Illumina adaptor sequences and a 9-bp barcode for library recognition (PE-III-PCR-F, 5′-AATGA TACGG CGACC ACCGA GATCT ACACT CTTTC CCTAC ACGAC GCTCT TCCGA TCT-3′; PE-III-PCR-001-096, 5′-CAAGC AGAAG ACGGC ATACG AGATN NNNNN NNNCG GTCTC GGCAT TCCTG CTGAA CCGCT CTTCC GATCT-3′, where N indicates the presence of a unique barcode).

Real-time qPCR before the first-step PCR was done to ensure uniform amplification, avoid overcycling templates, and to provide a basic estimate of bacterial biomass for each sample (i.e. total copies of the 16S gene per volume of DNA extraction from a single mouse fecal pellet). Both real-time and first-step PCRs were done similarly to the manufacturer’s protocol for Phusion polymerase (New England BioLabs, Ipswich, MA). For qPCR, reactions were assembled into 20 μL reaction volumes containing the following: DNA-free H_2_O, 8.9 μL; high fidelity (HF) buffer, 4 μL; dinucleotide triphosphates (dNTPs), 0.4 μL; PE16S_V4_U515_F (3 μM), 2 μL; PE16S_V4_E786_R (3 μM), 2 μL; BSA (20 mg/mL), 0.5 μL; EvaGreen (20×), 1 μL; Phusion, 0.2 μL; and template DNA, 1 μL. Reactions were cycled for 40 cycles with the following conditions: 98 °C for 2 min (initial denaturation); 40 cycles of 98 °C for 30 s (denaturation); 52 °C for 30 s (annealing); and 72 °C for 30 s (extension). Samples were diluted based on qPCR amplification to the level of the most dilute sample and amplified to the maximum number of cycles needed for PCR amplification of the most dilute sample (18 cycles, maximally, with no more than 8 cycles of second-step PCR). For first-step PCR, reactions were scaled (EvaGreen dye excluded; water increased) and divided into three 25-μL replicate reactions during both first- and second-step cycling reactions and cleaned after the first and second step using Agencourt AMPure XP-PCR purification (Beckman Coulter, Brea, CA) according to the manufacturer’s instructions. Second-step PCR contained the following: DNA-free H_2_O, 10.65 μL; HF buffer, 5 μL; dNTPs, 0.5 μL; PE-III-PCR-F (3 μM), 3.3 μL; PE-III-PCR-001-096 (3 μM), 3.3 μL; Phusion, 0.25 μL; and first-step PCR DNA, 2 μL. Reactions were cycled for 10 cycles with the following conditions: 98 °C for 30 s (initial denaturation); 10 cycles of 98 °C for 30 s (denaturation); 83 °C for 30 s (annealing); and 72 °C for 30 s (extension). Following second-step clean-up, product quality was verified by DNA gel electrophoresis and sample DNA concentrations determined using Quant-iT PicoGreen dsDNA Assay Kit (Thermo Fisher Scientific). The libraries were multiplexed together and sequenced using the paired-end with 250-bp paired-end reads approach on the MiSeq Illumina sequencing machine at the BioMicro Center (Massachusetts Institute of Technology, Cambridge, MA).

For the additional follow-up 16S samples from the seaweed experiment DNA was extracted using the AllPrep PowerFecal DNA/RNA Kit (Qiagen USA, Cat. No. 80244). DNA concentrations were determined using Quant-iT PicoGreen dsDNA Assay Kit (Thermo Fisher Scientific). Primary amplification was again performed with U515F and E786R targeting the V4 region of the 16S rRNA gene and reactions were cycled using the following conditions: 95 °C for 3 min (initial denaturation); 25 cycles of 95 °C for 30 s (denaturation); 55 °C for 30 s (annealing); and 72 °C for 30 s (extension). Correct amplification of the V4 region was verified with a Bioanalyzer (Agilent, USA). Indexing was performed using a custom (IDT, USA) 8nt dual indexing primer set (IDT-8nt-NXT_i5_9, 5′-AAT GAT ACG GCG ACC ACC GAG ATC TAC ACN NNN NNN NTC GTC GGC AGC G*T*C-3′; IDT-8nt-NXT_i7_13, 5′-CAA GCA GAA GAC GGC ATA CGA GAT NNN NNN NNG TCT CGT GGG CTC* G*G-3′, where N indicates the presence of a unique barcode and * indicates a phosphorothioate bond) following the indexing PCR supplied by the provider. Sequencing was performed with an Illumina NextSeq (NS500720) for 150 cycles at the Institute for Systems Biology sequencing core and demultiplexing was performed using bcl2fastq version 2.20.0.422.

Amplicon sequencing data were processed using DADA2 (ref. ^57^) and a custom 16S analysis pipeline available at https://github.com/gibbons-lab/mbtools. After performing general quality assessment, raw reads were filtered using the “filterAndTrim” method from DADA2 using a left trim of 10 bp to avoid low complexity 5′ sequences and a maximum of two expected errors per read under Illumina model. Length truncation was performed based on the quality profiles and ensuring that sufficient overlap for merging remained. Reads in the duration experiment were truncated at 240 and 150 bps for forward and reverse reads, respectively, and reads in the diet experiment were truncated at 240 and 170 bps. More than 88% of the reads in the duration experiment and 93% of the reads in the diet experiment passed quality filtering and were passed on to downstream processing with DADA2. Error rates were learned on a sample of 250 million bases and most of the inferred sequence variants could be merged across forward and reverse reads (>95% of preprocessed reads remaining). Less than 7% of all reads from both experiments were classified as chimeric and removed as well. Taxonomy was assigned to the sequence variants using the DADA2 Naive Bayes classifier with a bootstrap agreement of >50% and using the SILVA ribosomal database^58^ version 132. Species were assigned by exact alignment where possible. PERMANOVA was performed using the Bray–Curtis distance on rarefied read counts with the “adonis” function from the “vegan” package (https://CRAN.R-project.org/package=vegan). Amplicon sequence variants contributing to the separation of variances were identified from the coefficients of individuals regressions against the target variable (returned by the “adonis” function as well). Differential abundance tests for individual taxa on varying taxonomic ranks were performed with Beta-binomial likelihood ratio tests using the Corncob package^46^. False discovery rate was controlled by using the Benjamini–Hochberg correction^59^.

FASTQ files from the follow-up sequencing run on the seaweed samples generated at ISB were processed similarly as the previous 16S samples. However, because this protocol generated shorter reads (150 bp), only forward reads were used in the analysis. Sequencing depth for this set of follow-up samples was 2–3 orders of magnitude greater than the prior batch of samples.

### Power analysis

Power analysis for 16S amplicon data was performed by using a previously published independent data set of 48 control mice from the same laboratory and subjected to the same library prep, sequencing protocol, and data processing^37^. The resulting ASV tables were rarefied to an even depth of 10,000 sequences per sample during (two samples were dropped because they did not reach the 10K library depth, resulting in a final data set containing samples from 46 mice). ASV abundances were fit to beta-binomial distributions and the resulting proportions and overdispersion parameters were used to simulate new ASV abundance tables with predefined effect sizes of 1.1 to 10 (i.e. 10% to 1000% change) and per-group sample sizes (*n*) ranging from 4 to 64 (ref. ^46^). Only taxa with a fitted *μ* > 0.001 were considered here and abundance tables were injected with 50% true differentially abundant taxa for both Mann–Whitney and Corncob tests, and 10% true differentially abundant taxa for PERMANOVA. The sampled abundance tables were then used to calculate the true positive rate (TPR, or power) and FDR for Mann–Whitney *U*, beta-binomial LRTs and PERMANOVA. We aimed at obtaining at least 50% power/sensitivity while maintaining an FDR of 0.05 to ensure that we detect the majority of true-positive effects while observing fewer than 5% of false positives.

From the power curves shown in Fig. S1 we concluded that we can reliably detect PERMANOVA differences as low as an *R*^2^ of 0.05 and with a total *n* of 10 (5 samples per group). Mann–Whitney *U* and beta-binomial likelihood ratio tests could reliably detect differences with a fold change larger than twofold and with 20–30 samples (10–15 samples per group). Due to the fitted overdispersion parameters beta-binomial tests were generally more conservative than Mann–Whitney *U* tests (lower sensitivity) but provided better control of the FDR which decreased with increasing *n* for beta-binomial tests but increased slightly above the nominal value for Mann–Whitney tests. Consequently, we chose to use beta-binomial tests to prioritize control of the FDR over the false-negative rate.

Power analysis for metatranscriptomic data was performed using control mouse samples from a published study on an independent data set^46,60^. Raw sequencing data were processed with the same pipeline as used in this manuscript. Power and FDR were quantified by using the “powsimR” R package, which sampled new data based on negative binomial distributions^61^. Chosen ranges for log2-fold changes and sample size (*n*) were 0–5 and 4–100, respectively. Due to the pooled variance inference in DESeq2, differential expression could be detected with as few as six samples (three per group) with an average log2-fold change of 2.5 (uniform between 0 and 5) as long as the mean log2 expression of the transcript was at least 5 (i.e. 32 reads). All tests controlled the FDR well across all tested effect and samples sizes.

### Cefoperazone quantification using SRM

To extract cefoperazone from fecal samples of mice (collected on day 15, 27, 32, and 33), 750 µL of 90% acetonitrile/10% water and 50 µL internal standard (IS) ceftiofur at 50 µg/mL (dissolved in water) were added to each sample in a SK38 Soil Kit 2 mL tube (containing 0.1 mm glass beads, 1.4 mm ceramic (zirconium oxide) beads, and a 3 mm glass bead, Bertin Corp). Samples were disrupted at 4 °C using a Precellys 24 homogenizer (Bertin Corp) at 6500 r.p.m. for 3 × 30 s. Samples were centrifuged for 8 min at 16,000 rpm at 4 °C. The supernatant was removed, a 5 µL aliquot diluted 1:250 with 60% acetonitrile/40% water, and 5 µL injected for SRM analysis.

Extraction of cefoperazone from plasma was guided by Wu et al.^62^. Five hundred microliters acetonitrile, 12 µL water, and 38 µL IS ceftiofur at 0.4 µg/mL (dissolved in water) were added to 50 µL of plasma (collected on day 31). Samples were vortexed for 5 min, and then centrifuged for 8 min at 16,000 rpm at 4 °C. The supernatant was removed, an aliquot diluted 1:1 with Millipore water, and 5 µL injected for SRM analysis.

A 10-step dilution series of cefoperazone covering more than five orders of magnitude (262,144 fold range) with 8 µg/mL as the highest and 3.05 × 10^−5^ µg/mL as the lowest concentration on column was prepared in 60% water/40% acetonitrile. Each concentration was measured in technical triplicates.

Samples were analyzed with a 5500 QTRAP equipped with a Turbo Spray Ion Source (Sciex, Foster City, CA) and an 1290 Infinity HPLC system including a G4220A binary pump and column heater (Agilent Technologies Inc., Santa Clara, CA). Chromatographic separation was performed with a Zorbax SB-C18 analytical column (2.1 × 50 mm, 1.8 μm, Agilent) at 45 °C using 0.1% formic acid in water (A), 0.1% formic acid in acetonitrile (B), and a gradient from 30 to 75% B at 0.1–5 min, followed by a 0.5 min wash step at 100% B and equilibration with 100% A for 7.3 min at a flow rate of 0.2 mL/min. Samples were analyzed in SRM mode with Q1 and Q3 set to unit resolution, 100 ms dwell time and a 1.47 s cycle time. Ion spray voltage (IS) was set to 5500 V, temperature (TEM) to 500 °C, ion source gas 1 and 2 (GS1, GS2) to 35, curtain gas (CUR) to 35, and declustering potential (DP) to 70 eV. Data were acquired with Analyst 1.7 (Sciex). The most intense fragment ions for cefoperazone and ceftiofur were determined, the final method included six fragment ions for cefoperazone and eight ions for ceftiofur. Collision energies were optimized at 15 eV for cefoperazone and 25 eV for ceftiofur. Forty-four fecal and 15 plasma samples of mice were analyzed in three technical replicates.

Data were analyzed with Skyline 19.1.0.193 (ref. ^63^), peak integration was manually checked for each run. Quantification was performed with the most intense transition of 646.1/530.13 for cefoperazone using peak areas. SRM data and transitions used to measure cefoperazone and ceftiofur were deposited in the PeptideAtlas data repository and are available at http://www.peptideatlas.org/PASS/PASS01632.

### RNA extraction, RNA sequencing, and RNAseq data analysis

RNA was extracted from a total of 17 samples from the seaweed diet experiment with the AllPrep PowerFecal DNA/RNA Kit (Qiagen USA, Cat. No. 80244). The 17 samples included 7 target samples taken during antibiotic treatment (4 untreated and 3 non-responder) and 10 negative controls prior to antibiotic treatment (days 20 and 25, 6 responder and 4 non-responder). RNA integrity numbers (RIN) were obtained using a 2100 BioAnalyzer (Agilent USA) with the Eukaryote Total RNA Nano Series II chip (Agilent USA). The majority of samples showed RINs above 5 and samples with lower RIN (5 of the control samples) were included in sequencing while controlling for the effect of low integrity in downstream analyses by explicitly including RIN as a confounder, as described previously^64^. We attempted to isolate RNA from responder samples taken during antibiotic treatment, but RINs for these samples were much too low for library prep.

Ribosomal RNA was depleted from the 17 RNA-seq samples using the Ribo-Zero Gold rRNA Removal Kit (Illumina USA, Cat. No. MRZE724) and final concentrations were measured using the Qubit RNA HS Assay Kit (Thermo Fisher Scientific USA, Cat. No. Q32852). Library preparation was performed using the TruSeq Stranded mRNA LT Sample Prep Kit (Illumina USA, Cat. No. RS-122-2101) and all samples were sequenced in single end mode in one run on an Illumina NextSeq (NS500720) for 85 cycles, which yielded a total of 464 million reads.

Raw sequencing reads were quality filtered using the “filterAndTrim” function from DADA2 with a left trim of 5 bp and a maximum expected error (maxEE) of 1. More than 95% of the raw reads passed those filters and were used for all downstream analyses. No length truncation was performed due to the short length and high 3′ quality scores of the reads.

Transcripts were assembled de novo from the filtered reads with RNA Spades (version 3.12.0) across the full set of reads (http://cab.spbu.ru/software/rnaspades/)^65^ using the default parameters. Transcript abundances for each sample were quantified by aligning the filtered reads to the assembled transcripts with Bowtie2 version 2.3.4.3 (ref. ^66^). Mapping of unique reads to several transcripts was resolved by allowing up to 60 alternative alignments per read and counting the transcript abundances with an transcript length-aware Expectation-Maximization algorithm as used by Kallisto^67^.

Functional annotations for the de novo assembled transcripts were obtained by first aligning the transcripts to the M5NR database^49^ using DIAMOND version 0.9.21 (ref. ^68^). Functional annotations were then obtained by using the existing mapping between M5NR and the SEED subsystems database^50^ as downloaded from the MG-RAST FTP (ftp://ftp.metagenomics.anl.gov/data/misc/JGI/). Finally, abundances for functional groups were calculated by summing the reads for each unique SEED subsystem ID in each sample.

Normalization, differential abundance testing, and FDR adjustment for assembled transcripts or functional groups were performed using DESeq2 version 1.26.0 (ref. ^69^). To avoid a bimodal *p* value histogram, this was preceded by a prefiltering step removing features with an average abundances <10 reads or not appearing in at least two of the samples.

### Statistics and reproducibility

This study includes data from two independent experiments. The first experiment characterized the effect of treatment duration on the gut microbiome phenotype in a cohort of 20 mice distributed across five treatment groups (Fig. 1). All mice were singly housed and sampled independently. A total of 164 stool samples were obtained from those 20 mice during the course of the experiment. Of those, 20 samples were internal controls taken before the antibiotic treatments. Reported results are based on the remaining 144 samples. One of the samples did not reach our quality cutoff of 10,000 reads per sample after sequencing and was removed from a subset of the analyses, such as PCoAs and PERMANOVAs, leaving 143 samples for these analyses.

The second experiment aimed to confirm the phenotype and to associate it with microbial load in the gut. This experiment used a cohort of 28 mice distributed across two dietary regimes (14 each). Eight mice from each group were treated with antibiotics. All mice were single-housed and sampled independently. Mouse weights were measured at 17 time points during the duration of the study, yielding 476 weight measurements. A total of 73 fecal samples were obtained for 16S amplicon sequencing (Fig. 2). An additional 17 fecal samples were obtained for RNA-seq. In order to confirm some of the predictions in the second study, an additional 44 samples from 20 unique mice were subjected to 16S amplicon sequencing using a different protocol (see above). Another independent set of 44 fecal pellets and 15 blood samples were processed for cefoperazone quantification (Fig. S4).

Statistical analysis was performed as described in the “Methods” section. A power analysis using prior data not used in this study can be found in Fig. S1.

### Reporting summary

Further information on research design is available in the Nature Research Reporting Summary linked to this article.

## Supplementary information

Supplementary Information

Description of Additional Supplementary Files

Supplementary Data 1

Reporting Summary

## Data Availability

Raw sequencing data can be found in the Sequence Read Archive (SRA) (https://www.ncbi.nlm.nih.gov/sra) under the Bioproject accession numbers PRJNA525428, PRJNA525457, and PRJNA525684. SRM data and transitions used to measure cefoperazone and ceftiofur were deposited in the PeptideAtlas data repository and are available at http://www.peptideatlas.org/PASS/PASS01632. Source data underlying plots shown in figures are provided in Supplementary Data 1.

## Code availability

All workflows (as R notebooks), installation instructions and additional metadata are provided at https://github.com/gibbons-lab/mouse_antibiotics and allow reproduction of all results and figures from the manuscript starting from the raw data. More complex functionality including the used power analysis is provided in a dedicated R package (“mbtools”) which is provided along with documentation at https://github.com/gibbons-lab/mbtools.
