# Supplementary material for: Non-responder phenotype reveals apparent microbiome-wide antibiotic tolerance in the murine gut
Source: Commun Biol. 2021 Mar 9;4:316. doi: 10.1038/s42003-021-01841-8 (PMC7943787; doi:10.1038/s42003-021-01841-8)
Supplement: Supplementary file 1 — Supplementary Information [file 42003_2021_1841_MOESM1_ESM.pdf]

Supplemental Information

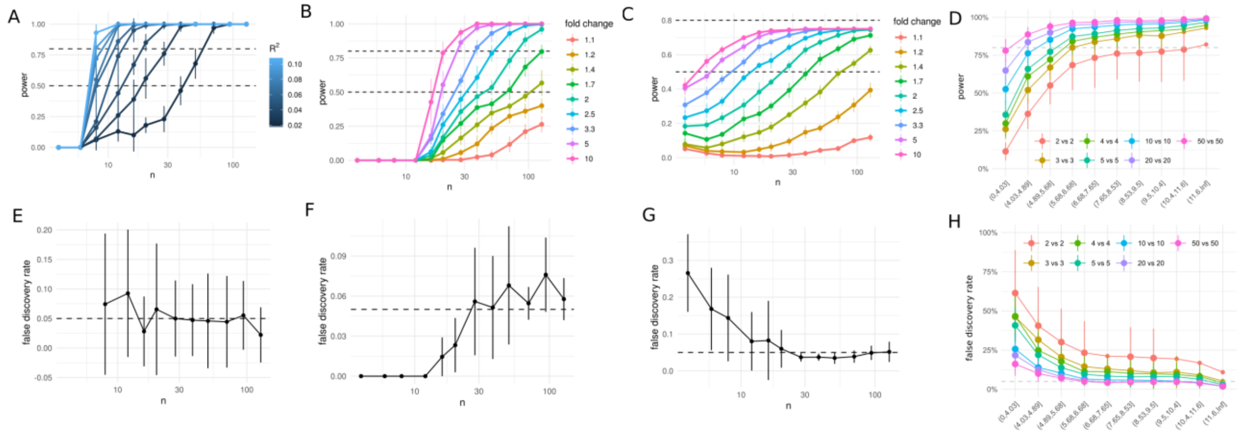

Figure S1. Power analysis (A) Power curves for PERMANOVA under varying R^2 and sample sizes (n). (B) Power curves for varying effect sizes (fold change) and sample sizes (n) for Mann-Whitney U tests. (C) Power curves for varying effect sizes (fold change) and sample sizes (n) for Corncob beta-binomial likelihood-ratio tests. (D) Power curves for varying sample sizes (n) and baseline expression using DESeq2. In (A-D) dashed lines denote a power of 0.5 and 0.8, respectively. (E-H) Denote the false discovery rate (FDR) as a function of sample size for PERMANOVA (E), Mann-Whitney U tests (F), Corncob (G), and DESeq2 (H). The dashed line in (E-H) denotes a FDR of 0.05.

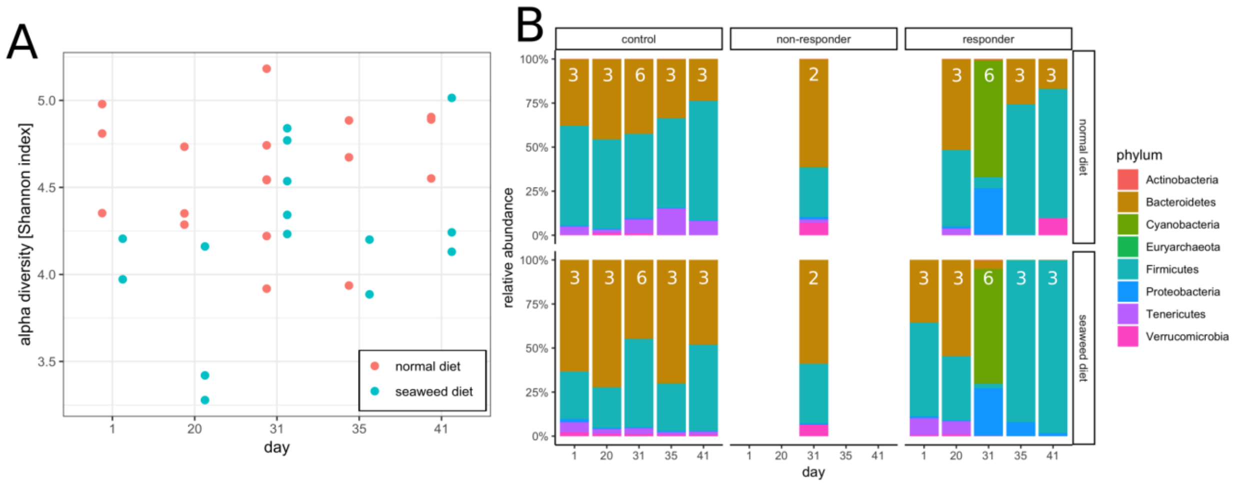

Figure S2. Alpha and beta-diversity trends for the diet study. (A) There were no significant differences in alpha-diversity (Shannon index) over time between control mice (no antibiotics) fed a normal diet and mice fed a 1% seaweed diet for 20 days (all Wilcoxon rank sum $p > 0.1$, n=30, for each day). All samples were rarefied to 10,000 reads. Shown are the 30/33 samples with more than 10,000 total reads per sample. (B) Stacked bars are means of at least 2 samples. Number of samples represented by each bar are shown as white insets. Community composition of control mice was slightly influenced by seaweed diet (7.7% explained variance, PERMANOVA $p = 0.01$) and this was mostly due to higher Bacteroides/Firmicutes ratio in mice fed the seaweed diet. Antibiotic treatment lasted from days 27-31. Responder mice showed few differences before treatment, but Bacteroidetes were completely lost in the seaweed-treated responder mice (8% explained variance, PERMANOVA $p = 0.01$). Non-responder mice did not show differences based on diet.

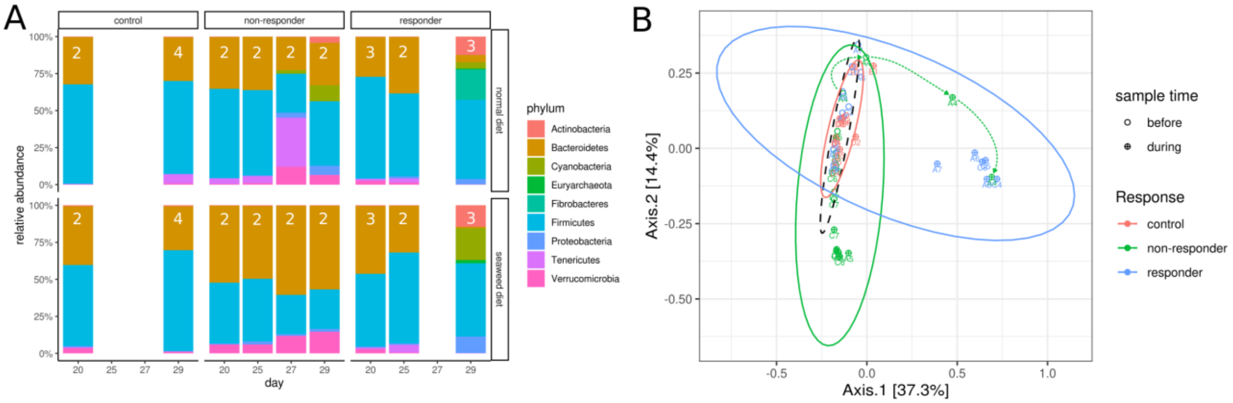

Figure S3. Additional validation samples for the seaweed experiment. (A) Relative phylum-level abundances for the 44 validation samples. Stacked bars are means of at least 2 samples. Number of samples represented by each bar are shown as white insets. (B) PCoA of 16S samples after diet (n=44, day >= 20). Symbol fill denotes sampling time relative to antibiotic treatment. Ellipses denote 95% confidence interval from a Student t-distribution. Dashed ellipsis denotes samples before antibiotic exposure. ASV abundances were rarefied to 20,000 reads for each sample. Percentages in brackets show explained variance by that axis. Labels on points denote individual mice (e.g. A1 is the first replicate mouse in treatment group A). Mouse A4 was designated a non-responder on day 31 (last day of antibiotic treatment), but on day 29 (also during antibiotic treatment) this mouse groups with the responders (B), which indicates that mice can transition from responder to non-responder status during antibiotic treatment. Dashed arrows denote the trajectory from day 20-29 for mouse A4.

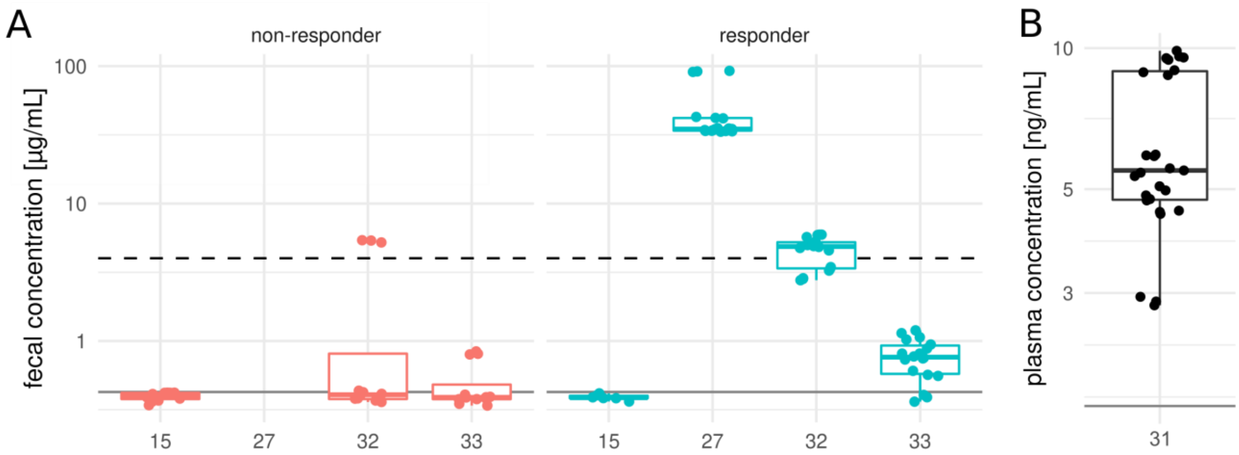

Figure S4. Quantification of cefoperazone in feces and plasma. Cefoperazone was quantified using selected reaction monitoring (SRM) with an internal standard of ceftiofur (see Materials and Methods). Antibiotic exposure was between day 27-31. The solid gray line denotes the maximum observed signal in untreated mice (background noise level). The dashed black line denotes the cefoperazone MIC_{50} for a panel of 357 anaerobic bacterial strains[48]. (A) Fecal cefoperazone concentrations for a subset of the mice with available fecal material. We used up all the available fecal pellets from non-responder mice during antibiotic treatment for generating 16S and metatranscriptomic data, which is why there is missing data for the non-responders on day 27. (B) Cefoperazone concentrations for a subset of mice with available plasma samples (i.e. cheek bleeds were taken from the first three replicate mice in each treatment group during antibiotic treatment; coincidentally, none of these replicate mice ended up being non-responders).

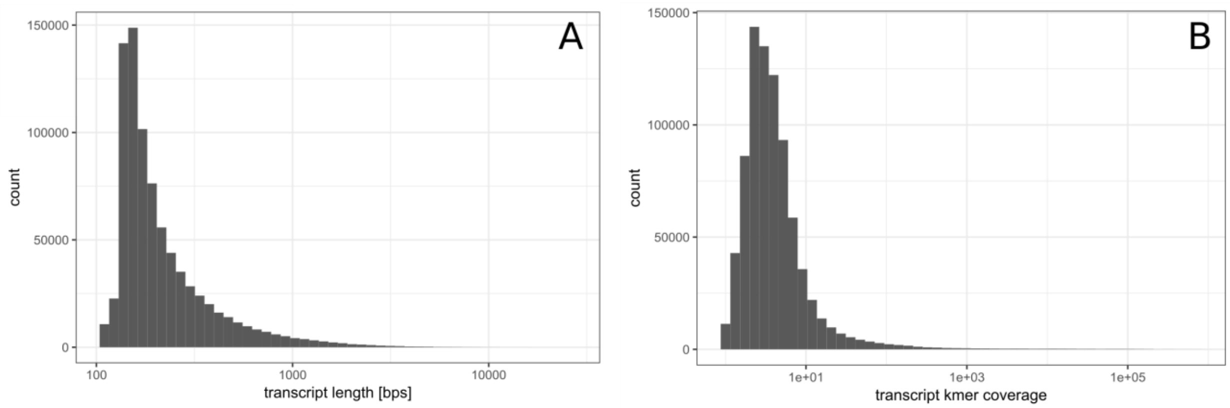

Figure S5. *De novo* assembly summaries for transcripts. (A) Length distribution of assembled transcripts. (B) Approximate coverage for assembled transcripts as estimated from k-mer coverage. Real coverage will always be larger than k-mer coverage.

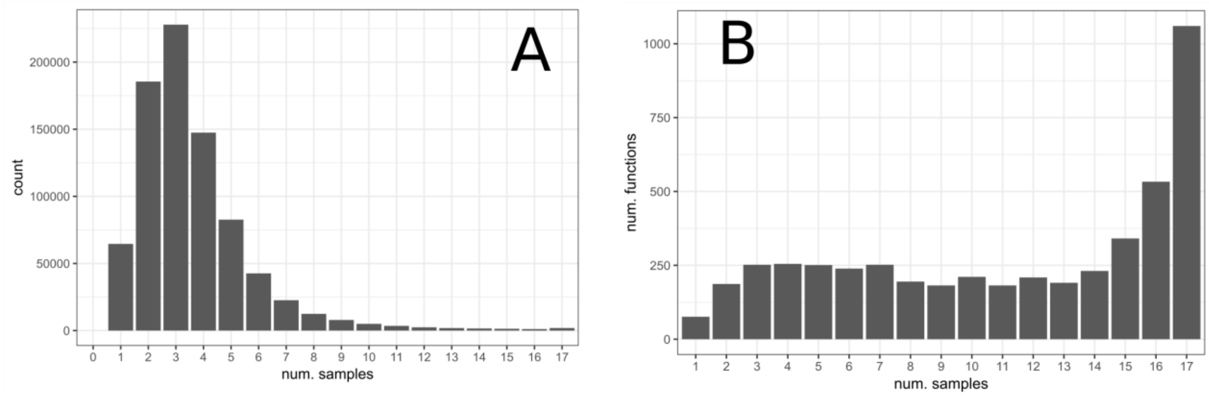

Figure S6. Distribution of transcripts and SEED clusters across RNA-seq samples. (A) Prevalence of assembled transcripts. Each bar denotes the number of transcripts observed in exactly k samples, where k is denoted on the x axis. (B) Prevalence of SEED clusters. Same as in A after collapsing transcripts to SEED clusters of orthologous proteins (unique IDs from the SEED database).
