# Supplementary material for: Non-responder phenotype reveals apparent microbiome-wide antibiotic tolerance in the murine gut
Source: Commun Biol. 2021 Mar 9;4:316. doi: 10.1038/s42003-021-01841-8 (PMC7943787; doi:10.1038/s42003-021-01841-8)
Supplement: Supplementary file 2 — Description of Additional Supplementary Files [file 42003_2021_1841_MOESM2_ESM.pdf]

Description of Additional Supplementary Files

File name: Supplementary Data 1

Description: The sheets in this file contain the source data for each of the main figures. For the exact commands to reproduce the figures, please refer to the Data Repository at https://github.com/gibbons-lab/mouse_antibiotics.
