# Supplementary material for: Non-responder phenotype reveals apparent microbiome-wide antibiotic tolerance in the murine gut
Source: Commun Biol. 2021 Mar 9;4:316. doi: 10.1038/s42003-021-01841-8 (PMC7943787; doi:10.1038/s42003-021-01841-8)
Supplement: Supplementary file 4 — Reporting Summary [file 42003_2021_1841_MOESM4_ESM.pdf]

nature research

Corresponding author(s): Sean M. Gibbons

Last updated by author(s): Dec 22, 2020

Reporting Summary

Nature Research wishes to improve the reproducibility of the work that we publish. This form provides structure for consistency and transparency in reporting. For further information on Nature Research policies, see our Editorial Policies and the Editorial Policy Checklist.

Statistics

For all statistical analyses, confirm that the following items are present in the figure legend, table legend, main text, or Methods section.

n/a	Confirmed	
☐	☒	The exact sample size (*n*) for each experimental group/condition, given as a discrete number and unit of measurement
☐	☒	A statement on whether measurements were taken from distinct samples or whether the same sample was measured repeatedly
☐	☒	The statistical test(s) used AND whether they are one- or two-sided<br>*Only common tests should be described solely by name; describe more complex techniques in the Methods section.*
☐	☒	A description of all covariates tested
☐	☒	A description of any assumptions or corrections, such as tests of normality and adjustment for multiple comparisons
☐	☒	A full description of the statistical parameters including central tendency (e.g. means) or other basic estimates (e.g. regression coefficient) AND variation (e.g. standard deviation) or associated estimates of uncertainty (e.g. confidence intervals)
☐	☒	For null hypothesis testing, the test statistic (e.g. *F*, *t*, *r*) with confidence intervals, effect sizes, degrees of freedom and *P* value noted<br>*Give P values as exact values whenever suitable.*
☒	☐	For Bayesian analysis, information on the choice of priors and Markov chain Monte Carlo settings
☒	☐	For hierarchical and complex designs, identification of the appropriate level for tests and full reporting of outcomes
☐	☒	Estimates of effect sizes (e.g. Cohen's *d*, Pearson's *r*), indicating how they were calculated

Our web collection on statistics for biologists contains articles on many of the points above.

Software and code

Policy information about availability of computer code

Data collection	Animal care<br>5- to 6-week-old female C57BL/6J mice ordered from Jackson Laboratories (Bar Harbor, ME) were housed and handled in Association for Assessment and Accreditation of Laboratory Animal Care (AAALAC)-accredited facilities using techniques and diets specifically approved by Massachusetts Institute of Technology's Committee on Animal Care (CAC) (MIT CAC protocol no. 0912-090-15 and 0909-090-18). The MIT CAC (Institutional Animal Care and Use Committee [IACUC]) specifically approved the studies as well as the single-housing and handling of these animals. Mice were euthanized using carbon dioxide at the end of the experiment.<br>Antibiotic duration experiment<br>For this 34-day experiment, 20 mice were assigned randomly and evenly to 5 treatment groups: control, 2 days of antibiotic exposure, 4 days of antibiotic exposure, 8 days of antibiotic exposure, and 16 days of antibiotic exposure. The β-lactam antibiotic cefoperazone was administered through drinking water at a concentration of 0.5 mg/mL, as in prior work33. Fecal samples were collected on the 2 days preceding antibiotic exposure, the last day of antibiotic exposure, and select timepoints following antibiotic exposure. Mice were weighed each sampling day. Fresh fecal samples were obtained within an hour of one another each day from all animals. Fecal samples were collected into 2 mL freezer tubes with 100 uL of anaerobic 40% glycerol containing 0.1% cysteine and transferred immediately to dry ice before being stored at -80° C prior to nucleic acid extraction.<br>Seaweed diet and antibiotic experiment<br>A new cohort of 28 mice were split randomly into two diet treatment groups and were fed with either a custom chow diet (Bio-Serv, Flemington NJ) containing 1% raw seaweed nori (Izumi Brand) or a standard control diet (product no. F3156; AN-93G; Bio-Serv, Flemington NJ). Prior to the experiment, animals were co-housed for 10 days and then singly housed for 7 days prior to separation into the seaweed treatment and control groups. After 20 days of dietary treatment, all mice resumed the standard diet. From day 26 to day 31, 8 mice from each diet group were administered 0.5 mg/mL cefoperazone in their drinking water as in the duration experiment. All mice were weighed and assessed daily. As per our IACUC protocol, any mouse showing significant signs of morbidity/suffering would be humanely euthanized. No mice showed signs of morbidity or distress during the course of both experiments. Fresh fecal samples were obtained within an hour of one another each day from all animals in all groups. Fecal samples were collected into anaerobic 40% glycerol containing 0.1% cysteine and

transferred immediately to dry ice before being stored at -80° C prior to nucleic acid extraction.

16S amplicon sequencing

DNA extractions

DNA from fecal samples and bacterial cultures was extracted using the MoBio High Throughput (HTP) PowerSoil Isolation Kit (MoBio Laboratories; now QIAGEN) with minor modifications. Briefly, samples were homogenized with bead beating and then 50 µL Proteinase K (QIAGEN) added, and samples were incubated in a 65°C water bath for 10 min. Samples were then incubated at 95°C for 10 min to deactivate the protease.

Amplicon sequencing library preparation and biomass quantification

Libraries for paired-end Illumina sequencing were constructed using a two-step 16S rRNA PCR amplicon approach as described previously with minor modifications56. The first-step primers (PE16S_V4_U515_F, 5′-ACACG ACGCT CTTCC GATCT YRYRG TGCCA GCMGC CGCGG TAA-3′; PE16S_V4_E786_R, 5′-CGGCA TTCCT GCTGA ACCGC TCTTC CGATC TGGAC TACHV GGGTW TCTAA T-3′) contain primers U515F and E786R targeting the V4 region of the 16S rRNA gene, as described previously56. Additionally, a complexity region in the forward primer (5′-YRYR-3′) was added to help the image-processing software used to detect distinct clusters during Illumina next-generation sequencing. A second-step priming site is also present in both the forward (5′-ACACG ACGCT CTTCC GATCT-3′) and reverse (5′-CGGCA TTCCT GCTGA ACCGC TCTTC CGATC T-3′) first-step primers. The second-step primers incorporate the Illumina adaptor sequences and a 9-bp barcode for library recognition (PE-III-PCR-F, 5′-AATGA TACGG CGACC ACCGA GATCT ACACT CTTTC CCTAC ACGAC GCTCT TCCGA TCT-3′; PE-III-PCR-001-096, 5′-CAAGC AGAAG ACGGC ATACG AGATN NNNNN NNNCG GTCTC GGCAT TCCTG CTGAA CCGCT CTTCC GATCT-3′, where N indicates the presence of a unique barcode).

Real-time qPCR before the first-step PCR was done to ensure uniform amplification, avoid overcycling templates, and to provide a basic estimate of bacterial biomass for each sample (i.e. total copies of the 16S gene per volume of DNA extraction from a single mouse fecal pellet). Both real-time and first-step PCRs were done similarly to the manufacturer's protocol for Phusion polymerase (New England BioLabs, Ipswich, MA). For qPCR, reactions were assembled into 20 µL reaction volumes containing the following: DNA-free H2O, 8.9 µL; high fidelity (HF) buffer, 4 µL; dinucleotide triphosphates (dNTPs), 0.4 µL; PE16S_V4_U515_F (3 µM), 2 µL; PE16S_V4_E786_R (3 µM), 2 µL; BSA (20 mg/mL), 0.5 µL; EvaGreen (20×), 1 µL; Phusion, 0.2 µL; and template DNA, 1 µL. Reactions were cycled for 40 cycles with the following conditions: 98°C for 2 min (initial denaturation); 40 cycles of 98°C for 30 s (denaturation); 52°C for 30 s (annealing); and 72°C for 30 s (extension). Samples were diluted based on qPCR amplification to the level of the most dilute sample and amplified to the maximum number of cycles needed for PCR amplification of the most dilute sample (18 cycles, maximally, with no more than 8 cycles of second-step PCR). For first-step PCR, reactions were scaled (EvaGreen dye excluded; water increased) and divided into three 25-µL replicate reactions during both first- and second-step cycling reactions and cleaned after the first and second step using Agencourt AMPure XP-PCR purification (Beckman Coulter, Brea, CA) according to manufacturer instructions. Second-step PCR contained the following: DNA-free H2O, 10.65 µL; HF buffer, 5 µL; dNTPs, 0.5 µL; PE-III-PCR-F (3 µM), 3.3 µL; PE-III-PCR-001-096 (3 µM), 3.3 µL; Phusion, 0.25 µL; and first-step PCR DNA, 2 µL. Reactions were cycled for 10 cycles with the following conditions: 98°C for 30 s (initial denaturation); 10 cycles of 98°C for 30 s (denaturation); 83°C for 30 s (annealing); and 72°C for 30 s (extension). Following second-step clean-up, product quality was verified by DNA gel electrophoresis and sample DNA concentrations determined using Quant-iT PicoGreen dsDNA Assay Kit (Thermo Fisher Scientific). The libraries were multiplexed together and sequenced using the paired-end with 250-bp paired-end reads approach on the MiSeq Illumina sequencing machine at the BioMicro Center (Massachusetts Institute of Technology, Cambridge, MA).

For the additional follow-up 16S samples from the seaweed experiment DNA was extracted using the AllPrep PowerFecal DNA/RNA Kit (Qiagen USA, Cat. No. 80244). DNA concentrations were determined using Quant-iT PicoGreen dsDNA Assay Kit (Thermo Fisher Scientific). Primary amplification was again performed with U515F and E786R targeting the V4 region of the 16S rRNA gene and reactions were cycled using the following conditions: 95°C for 3 min (initial denaturation); 25 cycles of 95°C for 30 s (denaturation); 55°C for 30 s (annealing); and 72°C for 30 s (extension). Correct amplification of the V4 region was verified with a Bioanalyzer (Agilent, USA). Indexing was performed using a custom (IDT, USA) 8nt dual indexing primer set (IDT-8nt-NXT_i5_9, 5′-AAT GAT ACG GCG ACC ACC GAG ATC TAC ACN NNN NNN NTC GTC GGC AGC G*T*C-3′; IDT-8nt-NXT_i7_13, 5′-CAA GCA GAA GAC GGC ATA CGA GAT NNN NNN NNG TCT CGT GGG CTC* G*G-3′, where N indicates the presence of a unique barcode and * indicates a phosphorothioate bond) following the indexing PCR supplied by the provider. Sequencing was performed with an Illumina NextSeq (NS500720) for 150 cycles at the Institute for Systems Biology sequencing core and demultiplexing was performed using bcl2fastq version 2.20.0.422.

16S amplicon sequencing data analysis

Amplicon sequencing data was processed using DADA257 and a custom 16S analysis pipeline available at https://github.com/gibbons-lab/mbtools. After performing general quality assessment, raw reads were filtered using the "filterAndTrim" method from DADA2 using a left trim of 10bp to avoid low complexity 5′ sequences and a maximum of 2 expected errors per read under Illumina model. Length truncation was performed based on the quality profiles and ensuring that sufficient overlap for merging remained. Reads in the duration experiment were truncated at 240 and 150 bps for forward and reverse reads respectively, and reads in the diet experiment were truncated at 240 and 170 bps. More than 88% of the reads in the duration experiment and 93% of the reads in the diet experiment passed quality filtering and were passed on to downstream processing with DADA2. Error rates were learned on a sample of 250 million bases and most of the inferred sequence variants could be merged across forward and reverse reads (>95% of preprocessed reads remaining). Less than 7% of all reads from both experiments were classified as chimeric and removed as well. Taxonomy was assigned to the sequence variants using the DADA2 Naive Bayes classifier with a bootstrap agreement of >50% and using the SILVA ribosomal database58 version 132. Species were assigned by exact alignment where possible. PERMANOVA was performed using the Bray-Curtis distance on rarefied read counts with the "adonis" function from the "vegan" package (https://CRAN.R-project.org/package=vegan). Amplicon sequence variants contributing to the separation of variances were identified from the coefficients of individuals regressions against the target variable (returned by the "adonis" function as well). Differential abundance tests for individual taxa on varying taxonomic ranks were performed with Beta-binomial likelihood ratio tests using the Corncob package46. False discovery rate was controlled by using the Benjamini-Hochberg correction59.

FASTQ files from the follow-up sequencing run on the seaweed samples generated at ISB were processed similarly as the previous 16S samples. However, because this protocol generated shorter reads (150bp), only forward reads were used in the analysis. Sequencing depth for this set of follow-up samples was 2-3 orders of magnitude greater than the prior batch of samples.

Power analysis

Power analysis for 16S amplicon data was performed by using a previously published independent data set of 48 control mice from the same laboratory and subjected to the same library prep, sequencing protocol and data processing 37. The resulting ASV tables were rarefied to an even depth of 10,000 sequences per sample during (two samples were dropped because they did not reach the 10K library depth, resulting in a final data set containing samples from 46 mice). ASV abundances were fit to beta-binomial distributions and the resulting proportions and overdispersion parameters were used to simulate new ASV abundance tables with predefined effect sizes of 1.1 to 10 (i.e. 10% to 1000% change) and per-group sample sizes (n) ranging from 4 to 64 46. Only taxa with a fitted $\mu > 0.001$ were considered here and abundance tables were injected with 50% true differentially abundant taxa for both Mann-Whitney and Corncob tests, and 10% true differentially abundant taxa for PERMANOVA. The sampled abundance tables were then used to calculate the true positive rate (TPR, or power) and false discovery rate

(FDR) for Mann-Whitney U, Beta-binomial LRTs and PERMANOVA. We aimed at obtaining at least 50% power/sensitivity while maintaining a FDR of 0.05 to ensure that we detect the majority of true positive effects while observing fewer than 5% of false positives.

From the power curves shown in Fig. S1 we concluded that we can reliably detect PERMANOVA differences as low as an R2 of 0.05 and with a total n of 10 (5 samples per group). Mann-Whitney U and beta-binomial likelihood ratio tests could reliably detect differences with a fold-change larger than 2-fold and with 20-30 samples (10-15 samples per group). Due to the fitted overdispersion parameters beta-binomial tests were generally more conservative than Mann-Whitney U tests (lower sensitivity) but provided better control of the false discovery rate which decreased with increasing n for beta-binomial tests but increased slightly above the nominal value for Mann-Whitney tests. Consequently, we chose to use beta-binomial tests to prioritize control of the false discovery rate over the false negative rate.

Power analysis for metatranscriptomic data was performed using control mouse samples from a published study on an independent data set 46,60. Raw sequencing data was processed with the same pipeline as used in this manuscript. Power and FDR were quantified by using the "powsimR" R package, which sampled new data based on negative binomial distributions61. Chosen ranges for log2-fold changes and sample size (n) were 0 to 5 and 4 to 100, respectively. Due to the pooled variance inference in DESeq2, differential expression could be detected with as few as 6 samples (3 per group) with an average log2-fold change of 2.5 (uniform between 0-5) as long as the mean log2 expression of the transcript was at least 5 (i.e. 32 reads).

All tests controlled the false discovery rate well across all tested effect and samples sizes.

Cefoperazone quantification using Selected Reaction Monitoring (SRM)

Mice fecal sample preparation

To extract cefoperazone from fecal samples of mice (collected on day 15, 27, 32 and 33), 750 µL of 90% acetonitrile/10% water and 50 µL internal standard (IS) ceftiofur at 50 µg/mL (dissolved in water) were added to each sample in a SK38 Soil Kit 2mL tube (containing 0.1mm glass beads, 1.4mm ceramic (zirconium oxide) beads and a 3mm glass bead, Bertin Corp). Samples were disrupted at 4°C using a Precellys 24 homogenizer (Bertin Corp) at 6500 rpm for 3 x 30 s. Samples were centrifuged for 8 min at 16,000 rpm at 4°C. The supernatant was removed, a 5 µL aliquot diluted 1:250 with 60% acetonitrile/40% water, and 5 µL injected for SRM analysis.

Mice plasma sample preparation

Extraction of cefoperazone from plasma was guided by Wu et al. 62. 500 µL acetonitrile, 12 µL water and 38 µL IS ceftiofur at 0.4 µg/mL (dissolved in water) were added to 50 µL of plasma (collected on day 31). Samples were vortexed for 5 min, then centrifuged for 8 min at 16,000 rpm at 4°C. The supernatant was removed, an aliquot diluted 1:1 with Millipore water, and 5 µL injected for SRM analysis.

Dilution curve for cefoperazone

A 10-step dilution series of cefoperazone covering more than 5 orders of magnitude (262,144 fold range) with 8 µg/mL as the highest and 3.05*10-5 µg/mL as the lowest concentration on column was prepared in 60% water/40% acetonitrile. Each concentration was measured in technical triplicates.

Selected Reaction Monitoring (SRM) analysis

Samples were analyzed with a 5500 QTRAP equipped with a Turbo Spray Ion Source (Sciex, Foster City, CA) and an 1290 Infinity HPLC system including a G4220A binary pump and column heater (Agilent Technologies Inc., Santa Clara, CA). Chromatographic separation was performed with a Zorbax SB-C18 analytical column (2.1 x 50mm, 1.8 µm, Agilent) at 45°C using 0.1% formic acid in water (A), 0.1% formic acid in acetonitrile (B), and a gradient from 30% to 75% B at 0.1-5 min, followed by a 0.5 min wash step at 100% B and equilibration with 100% A for 7.3 min at a flow rate of 0.2 mL/min. Samples were analyzed in SRM mode with Q1 and Q3 set to unit resolution, 100 ms dwell time and a 1.47 s cycle time. Ion spray voltage (IS) was set to 5500 V, temperature (TEM) to 500°C, ion source gas 1 and 2 (GS1, GS2) to 35, curtain gas (CUR) to 35, and declustering potential (DP) to 70 eV. Data was acquired with Analyst 1.7 (Sciex). The most intense fragment ions for cefoperazone and ceftiofur were determined, the final method included six fragment ions for cefoperazone and eight ions for ceftiofur. Collision energies were optimized at 15 eV for cefoperazone and 25 eV for ceftiofur. 44 fecal and 15 plasma samples of mice were analyzed in three technical replicates.

SRM data analysis

Data was analyzed with Skyline 19.1.0.193 63, peak integration was manually checked for each run. Quantification was performed with the most intense transition of 646.1/530.13 for cefoperazone using peak areas. SRM data and transitions used to measure cefoperazone and ceftiofur were deposited in the PeptideAtlas data repository and are available at http://www.peptideatlas.org/PASS/PASS01632.

RNA extraction, RNA sequencing, and RNAseq data analysis

RNA extraction

RNA was extracted from a total of 17 samples from the seaweed diet experiment with the AllPrep PowerFecal DNA/RNA Kit (Qiagen USA, Cat. No. 80244). The 17 samples included 7 target samples taken during antibiotic treatment (4 untreated and 3 non-responder) and 10 negative controls prior to antibiotic treatment (day 20 and 25, 6 responder and 4 non-responder). RNA integrity numbers (RIN) were obtained using a 2100 BioAnalyzer (Agilent USA) with the Eukaryote Total RNA Nano Series II chip (Agilent USA). The majority of samples showed RINs above 5 and samples with lower RIN (5 of the control samples) were included in sequencing while controlling for the effect of low integrity in downstream analyses by explicitly including RIN as a confounder, as described previously64. We attempted to isolate RNA from responder samples taken during antibiotic treatment, but RINs for these samples were much too low for library prep.

Library preparation and RNA sequencing

Ribosomal RNA was depleted from the 17 RNA-seq samples using the Ribo-Zero Gold rRNA Removal Kit (Illumina USA, Cat. No. MRZE724) and final concentrations were measured using the Qubit RNA HS Assay Kit (ThermoFisher Scientific USA, Cat. No. Q32852). Library preparation was performed using the TruSeq Stranded mRNA LT Sample Prep Kit (Illumina USA, Cat. No. RS-122-2101) and all samples were sequenced in single end mode in one run on an Illumina NextSeq (NS500720) for 85 cycles, which yielded a total of 464 million reads.

RNA-seq data analysis

Raw sequencing reads were quality filtered using the "filterAndTrim" function from DADA2 with a left trim of 5bp and a maximum expected error (maxEE) of 1. More than 95% of the raw reads passed those filters and were used for all downstream analyses. No length truncation was performed due to the short length and high 3' quality scores of the reads.

Transcripts were assembled de novo from the filtered reads with RNA Spades (version 3.12.0) across the full set of reads (http://cab.spbu.ru/software/rnaspades/)65 using the default parameters. Transcript abundances for each sample were quantified by aligning the filtered reads to the assembled transcripts with Bowtie2 version 2.3.4.366. Mapping of unique reads to several transcripts was resolved by allowing up to 60 alternative alignments per read and counting the transcript abundances with an transcript length-aware Expectation-Maximization algorithm as used by Kallisto67.

Functional annotations for the de novo assembled transcripts were obtained by first aligning the transcripts to the M5NR database49 using

	DIAMOND version 0.9.2168. Functional annotations were then obtained by using the existing mapping between M5NR and the SEED subsystems database50 as downloaded from the MG-RAST FTP (ftp://ftp.metagenomics.anl.gov/data/misc/JGI/). Finally, abundances for functional groups were calculated by summing the reads for each unique SEED subsystem ID in each sample.<br>Normalization, differential abundance testing and false discovery rate (FDR) adjustment for assembled transcripts or functional groups were performed using DESeq2 version 1.26.069. To avoid a bimodal p-value histogram, this was preceded by a prefiltering step removing features with an average abundances <10 reads or not appearing in at least two of the samples.
Data analysis	All workflows (as R notebooks), installation instructions and additional metadata are provided at https://github.com/gibbons-lab/mouse_antibiotics and allow reproduction of all results and figures from the manuscript starting from the raw data. More complex functionality including the used power analysis is provided in a dedicated R package ("mbtools") which is provided along with documentation at https://github.com/gibbons-lab/mbtools.

For manuscripts utilizing custom algorithms or software that are central to the research but not yet described in published literature, software must be made available to editors and reviewers. We strongly encourage code deposition in a community repository (e.g. GitHub). See the Nature Research guidelines for submitting code & software for further information.

Data

Policy information about availability of data

All manuscripts must include a data availability statement. This statement should provide the following information, where applicable:

- Accession codes, unique identifiers, or web links for publicly available datasets
- A list of figures that have associated raw data
- A description of any restrictions on data availability

Raw sequencing data can be found in the Sequence Read Archive (SRA) (https://www.ncbi.nlm.nih.gov/sra) under the Bioproject accession numbers PRJNA525428, PRJNA525457, and PRJNA525684. SRM data and transitions used to measure cefoperazone and ceftiofur were deposited in the PeptideAtlas data repository and are available at http://www.peptideatlas.org/PASS/PASS01632.

Field-specific reporting

Please select the one below that is the best fit for your research. If you are not sure, read the appropriate sections before making your selection.

☒ Life sciences ☐ Behavioural & social sciences ☐ Ecological, evolutionary & environmental sciences

For a reference copy of the document with all sections, see nature.com/documents/nr-reporting-summary-flat.pdf

Life sciences study design

All studies must disclose on these points even when the disclosure is negative.

Sample size	Antibiotic duration experiment<br>For this 34-day experiment, 20 mice were assigned randomly and evenly to 5 treatment groups: control, 2 days of antibiotic exposure, 4 days of antibiotic exposure, 8 days of antibiotic exposure, and 16 days of antibiotic exposure.<br><br>A new cohort of 28 mice were split randomly into two diet treatment groups and were fed with either a custom chow diet (Bio-Serv, Flemington NJ) containing 1% raw seaweed nori (Izumi Brand) or a standard control diet (product no. F3156; AN-93G; Bio-Serv, Flemington NJ).
Data exclusions	A handful of samples that failed to generate enough sequence data were excluded from downstream analyses (see Methods section).
Replication	We ran two independent mouse experiments, to replicate our major observation of non-responder mice.
Randomization	Mice were randomly assigned to treatment groups.
Blinding	Investigators were not blind to which mice belonged to which treatment group. However, investigators were blind to which mice were 'non-responders' during the experiment (i.e. we were only able to determine responder status from the sequencing data, after the experiment was complete).

Reporting for specific materials, systems and methods

We require information from authors about some types of materials, experimental systems and methods used in many studies. Here, indicate whether each material, system or method listed is relevant to your study. If you are not sure if a list item applies to your research, read the appropriate section before selecting a response.

Materials & experimental systems

n/a	Involved in the study
☒	☐ Antibodies
☒	☐ Eukaryotic cell lines
☒	☐ Palaeontology and archaeology
☐	☒ Animals and other organisms
☒	☐ Human research participants
☒	☐ Clinical data
☒	☐ Dual use research of concern

Methods

n/a	Involved in the study
☒	☐ ChIP-seq
☒	☐ Flow cytometry
☒	☐ MRI-based neuroimaging

Animals and other organisms

Policy information about studies involving animals; ARRIVE guidelines recommended for reporting animal research

Laboratory animals	C57BL/6J mice ordered from Jackson Laboratories (Bar Harbor, ME)
Wild animals	NA
Field-collected samples	NA
Ethics oversight	Mice were housed and handled in Association for Assessment and Accreditation of Laboratory Animal Care (AAALAC)-accredited facilities using techniques and diets specifically approved by Massachusetts Institute of Technology's Committee on Animal Care (CAC) (MIT CAC protocol no. 0912-090-15 and 0909-090-18). The MIT CAC (Institutional Animal Care and Use Committee [IACUC]) specifically approved the studies as well as the single-housing and handling of these animals. Mice were euthanized using carbon dioxide at the end of the experiment.

Note that full information on the approval of the study protocol must also be provided in the manuscript.
